# Synthesis of New Chromene Derivatives Targeting Triple-Negative Breast Cancer Cells

**DOI:** 10.3390/cancers15102682

**Published:** 2023-05-09

**Authors:** Aysha Alneyadi, Zohra Nausheen Nizami, Hanan E. Aburawi, Soleiman Hisaindee, Muhammad Nawaz, Samir Attoub, Gaber Ramadan, Nehla Benhalilou, Mazoun Al Azzani, Yassine Elmahi, Aysha Almeqbali, Khalid Muhammad, Ali H. Eid, Ranjit Vijayan, Rabah Iratni

**Affiliations:** 1 Department of Biology, College of Science, United Arab Emirates University, Al Ain P.O. Box 15551, United Arab Emirates; 2 Department of Chemistry, College of Science, United Arab Emirates University, Al Ain P.O. Box 15551, United Arab Emirates; 3 Department of Nano-Medicine Research, Institute for Research and Medical Consultations (IRMC), Imam Abdulrahman Bin Faisal University, P.O. Box 1982, Dammam 31441, Saudi Arabia; 4 Department of Pharmacology & Therapeutics, College of Medicine & Health Sciences, United Arab Emirates University, Al-Ain P.O. Box 15551, United Arab Emirates; 5 Department of Basic Medical Sciences, College of Medicine, QU Health, Qatar University, Doha P.O. Box 2713, Qatar

**Keywords:** microtubules, senescence, chromenes, mitotic slippage, multinucleation, apoptosis

## Abstract

**Simple Summary:**

To date, breast cancer remains a leading cause of cancer-related deaths among women. The triple-negative breast cancer (TNBC) subtype is the most aggressive and invasive form, and it frequently develops resistance to chemotherapy. Hormonal therapy can also be ineffective against TNBC. Herein, we synthesized two new chromene compounds and tested their activity against hormone-responsive and TNBC cells. We found that both compounds specifically inhibited the proliferation of TNBC cells with little or no effect on hormone-responsive cells. We found that our compounds disrupted the polymerization of microtubules within the cytoskeleton, which are required for proper cell division. Consequently, the TNBC cells underwent permanent growth arrest and cell death. Moreover, the chromene compounds efficiently blocked TNBC migration and inhibited the formation of blood vessels; these are two events crucial for cancer metastasis. Our findings highlight the therapeutic potential of these compounds against TNBC.

**Abstract:**

Breast cancer continues to be the leading cause of cancer-related deaths among women worldwide. The most aggressive type of breast cancer is triple-negative breast cancer (TNBC). Indeed, not only does TNBC not respond well to several chemotherapeutic agents, but it also frequently develops resistance to various anti-cancer drugs, including taxane mitotic inhibitors. This necessitates the search for newer, more efficacious drugs. In this study, we synthesized two novel chromene derivatives (**C1** and **C2**) and tested their efficacy against a battery of luminal type A and TNBC cell lines. Our results show that **C1** and **C2** significantly and specifically inhibited TNBC cell viability but had no effect on the luminal A cell type. In addition, these novel compounds induced mitotic arrest, cell multinucleation leading to senescence, and apoptotic cell death through the activation of the extrinsic pathway. We also showed that the underlying mechanisms for these actions of **C1** and **C2** involved inhibition of microtubule polymerization and disruption of the F-actin cytoskeleton. Furthermore, both compounds significantly attenuated migration of TNBC cells and inhibited angiogenesis in vitro. Finally, we performed an *in silico* analysis, which revealed that these novel variants bind to the colchicine binding site in β-tubulin. Taken together, our data highlight the potential chemotherapeutic properties of two novel chromene compounds against TNBC.

## 1. Introduction

Breast cancer remains the world’s most prevalent cancer type and is one of the leading causes of cancer-related mortality [1]. In 2020, approximately 2.3 million women were diagnosed with breast cancer and of these, 684,996 died, representing approximately 16% of all cancer deaths in women worldwide [1,2]. Moreover, the occurrence rate of breast cancer is constantly increasing, with over three million new cases and one million associated deaths predicted by 2040 [1].

Breast cancer is a highly heterogeneous disease both clinically and genetically. It can be classified into four subtypes based on the immunohistochemical expression of the following hormone receptors: human epidermal growth factor receptor (HER2), progesterone receptor (PR), and estrogen receptor (ER) [3] as luminal A, luminal HER2-positive, and triple-negative breast cancer (TNBC) [3]. Compared to other subtypes, TNBC, which lacks expression of all three receptors, is characterized by a high proliferative index, high invasiveness, poor prognosis, early relapse, and a tendency to be diagnosed in advanced stages [3,4,5].

The three aforementioned hormone receptors are frequently targeted biomarkers in breast cancer treatment, and absence of their expression makes conventional chemotherapeutic and hormonal therapies relatively ineffective against TNBC. This makes TNBC the most aggressive and lethal subtype of breast cancer [6]. TNBC can evade apoptosis through its resistance to several cytotoxic drugs used to treat it, including cyclophosphamide, doxorubicin, and paclitaxel [7,8]. In addition, many agents used to treat cancers, including TNBC, have been shown to have major drawbacks in terms of drug resistance, adverse effects, and toxicity [9]. Thus, there is a need to discover and develop novel anticancer agents that can specifically target TNBC.

**Figure 1:**
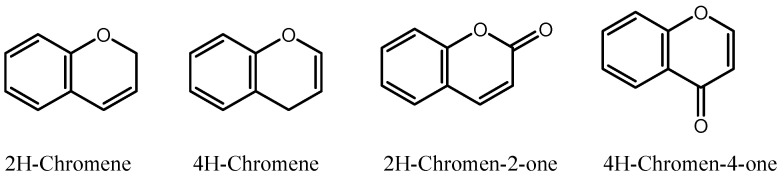
Chromene motifs in natural products.

Chromenes refer to a class of bicyclic oxygen-containing heterocycles with a fused benzene ring. They are natural compounds that are widely distributed in various plant species, with four common and readily identifiable structural motifs, namely 2H-chromene, 4H-chromene, 2H-chromene-2-one, and 4H-chromen-4-one [10] (Figure 1). The 4H-chromenes, in particular, are emerging as potent novel anticancer agents [11,12]. Their anticancer activity has been observed in many natural compounds, such as tephrosin against lung and pancreatic cancer [13,14], calanone against cervical carcinoma [15], acronycine against ovarian, lung, and colon cancer [16], and seselin against skin cancer [17].

The structure of the chromenes offers advantages that make them appealing candidates for the development of new anticancer drugs. They are easily accessible through a single-step three component condensation reaction comprising of a phenol, a methylene-active derivative, and aryl aldehydes. Moreover, the products are crystalline and easily purified following bulk synthesis. In addition, the chromene scaffold offers unique opportunities for the introduction of functional groups that can influence their biological activity against cancer.

Several modified 4H-chromenes were shown to be effective, albeit at nanomolar concentrations, against various human cancers [18,19,20,21,22]. In some cases, they were found to be effective even against chemo-resistant cancer such as multi-drug resistant HL60/MX22 [22] and cisplatin resistant ovarian cancer such as OVCAR-3, A2780-cisR, and TOV112D [23].

The molecular mechanism of the action of these 4H-chromenes against various types of human cancers does not seem to be restricted to one mechanism, and involves several pathways that include apoptosis, cell cycle arrest, nuclear fragmentation, microtubule depolarization, and tumor vasculature disruption [24,25]. The 4-Aryl-4H-chromene-3-carbonitrile derivatives, for example, were reported to effectively inhibit Src kinases, which are overexpressed in human colorectal and leukemia cell lines [26]. Crolibulin, another 4H-chromene derivative, (currently in Phase I/II clinical trials for the treatment of advanced solid tumors) induces depolarization of the microtubules and disruption of tumor vasculature [24]. These findings suggest that 4H-chromene compounds are promising candidates for cancer therapy as they are easily accessible, and they operate against a wide range of targets, thus having the ability to prevent the development of drug-resistant cancers such as TNBC.

In this paper, we synthesized two new variants of 4H-chromene compounds, **C1** and **C2**, and tested their anticancer activity against four cell lines, including two luminal A and two TNBC cancer cell lines.

## 2. Materials and Methods

### 2.1. Cell Culture and Reagents

Human breast cancer cells MDA-MB-231(300275), MCF-7 (300273), and T47D (300353) were obtained from Cell Line Service, Eppelheim, Germany, and Hs578T was obtained from ATCC. MDA-MB-231, Hs578T, and MCF-7 were maintained in DMEM (11995073; Gibco/Thermo Fisher Scientific, Waltham, MA, USA), whereas T47D was maintained in RPMI (1770766; Gibco, Waltham, MA, USA). Culture media were supplemented with fetal bovine serum (10%) (A4766801; Gibco) and 100 U/mL penicillin/streptomycin/glutamine (2321073; Gibco). Human umbilical vein endothelial cells (HUVECs; Millipore, Burlington, MA, USA) were cultured in an EndoGROTM basal medium supplemented with an EndoGRO-VEGF supplement kit (SCME-002S, Merck Millipore, Burlington, MA, USA).

Antibodies against p27 (3686), caspase 8 (9746), anti-mouse IgG-HRP (7076P2), and anti-rabbit IgG-HRP (7074S) were obtained from Cell Signaling (Danvers, MA, USA). Antibodies against α-tubulin (SC-53646), β-tubulin (SC-5274), and β-actin (sc47778) were obtained from Santa Cruz Biotechnology, Inc. (Dallas, TX, USA). Antibodies against H3pSer10 (51336), γH2AX (07-164), and p21 (05-655) were obtained from Millipore. Finally, an antibody against cleaved PARP was obtained from Abcam (Ab9739) (Cambridge, UK) and an antibody against p16 (554079) was obtained from BD Pharmingen (Franklin Lakes, NJ, USA).

### 2.2. Measurement of Cell Viability

Five thousand cells from MDA-MB-231, Hs578T, T47D, and MCF-7 were seeded in triplicate in 96-well cell culture plates. After 24 h of culture, cells were treated with various concentrations of chromenes **C1** and **C2** or a control. Cell viability was assessed 24 and 48 h post-treatment using an MTT assay kit (ab 211091; Abcam) with all procedures performed as per the manufacturer’s specifications.

The cellular viability was further assessed by using a method that distinguished between viable and dead cells based on their selective permeability to two DNA binding dyes. Cell viability was carried out as previously described [27] using the Muse^®^ Count & Viability Kit. Briefly, on the day of treatment, considered as day 0, cells were counted to determine the number of cells per well before adding chromene **C1** and **C2**. Viable cells were determined using the Muse™ Cell Analyzer as described by the manufacturer (Luminex Corporation, Austin, TX, USA).

### 2.3. Colony Growth Assay

TNBC cells (250 per well) were cultured in a 6-well cell culture plate for 3 days before adding freshly prepared growth medium with or without various concentrations of chromene **C1** or **C2**. Cells were then grown for 8–9 days to allow for the formation of colonies. Colonies were then washed with PBS, fixed with formalin solution (10%), and stained with crystal violet.

### 2.4. Cell Cycle Analysis

Cell cycle distributions of untreated and chromene **C1**- and **C2**-treated MDA-MB-231 and Hs578T cells were analyzed using a Muse™ Cell Cycle Kit (Millipore), with all procedures following the instructions provided by the manufacturer. Cells grown in 6 cm dishes were treated with or without **C1** and **C2** for 24 h. Cell cycle analysis was then performed as previously described [27], using the Muse™ Cell Analyzer. All data collected were analyzed using FlowJo version 7.6.5.

### 2.5. Caspase 3/7 Activity

Samples of 5000 MB-231 cells seeded into 96-well cell culture plates were treated with or without chromene **C1** or **C2** for 24 h. Caspase 3 and 7 activity was then measured using a luminescent assay kit (Promega Corporation, Madison, WI, USA), with all procedures performed as per the manufacturer’s instructions.

### 2.6. Immunofluorescence Staining

MDA-MB-231 cells (4 × 10^4^) were cultured in a two-well chamber slide for 24 h, then exposed to chromene **C1** or **C2** for 24 h. Next, the cells were fixed with formalin (10), permeabilized with Triton X-100 (0.1%), preincubated with 5% non-fat dry milk, and finally incubated with α-tubulin overnight at 4 °C. After washing with PBS, the cells were incubated with a rhodamine-conjugated secondary antibody, washed again with PBS, and their DNA was stained with 4′, 6-diamine-2-phenylindole dihydrochloride (DAPI). Actin filaments in control and treated cells were visualized as follows: after fixation and permeabilization as described above, the cells were stained with rhodamine-conjugated phalloidin followed by DAPI staining. Fluorescence was visualized using a fluorescent microscope CKX53 (Olympus, Tokyo, Japan).

### 2.7. Hematoxylin and Eosin Staining

MDA-MB-231 and Hs 578T cells (4 × 10^4^) were cultured on a 2-well chamber slide for 24 h, then exposed to chromene **C1** or **C2** for 24 h. Cells were washed twice with PBS and fixed with 3.7% glutaraldehyde for 20 min at room temperature. After fixation, cells were washed again three times with PBS and permeabilized with Triton X-100 (0.2%). Cells were washed again 3 times with PBS and then with distilled water. Cells were then stained with hematoxylin for 15 min and washed with distilled water for 2 min to remove excess stain before staining with eosin solution for 5 min. Stained cells were observed under a light microscope.

### 2.8. Whole-Cell Extraction and Western Blotting Analysis

MDA-MB-231 and Hs578T cells (2.8  × 10^6^) were seeded in 100 mm culture dishes and cultured for 24 h prior to treatment with chromene **C1** or **C2** at the indicated concentrations and for the listed incubation times. Following treatment, protein extraction, protein concentration quantification, and protein detection by Western blotting were conducted as previously described [27]. Protein expression was analyzed by detection of chemiluminescence using a Li-COR C-DiGit blot scanner. Densitometry quantification of the Western blots was carried out using ImageJ software. Original blots can be found at Supplementary File S2.

### 2.9. Senescence-Associated β-Galactosidase Staining

TNBC cells were seeded (2.5 × 10^5^ cells/dish) in 60 mm culture dishes for 24 h and treated with DMSO (control) or chromene **C1** or **C2** for 48 h. Cells were then washed with PBS and fixed with 2% formaldehyde/0.2% glutaraldehyde. The activity of β-galactosidase was then quantified using a previously described method [28].

### 2.10. Wound-Healing Assay

The effect of **C1** and **C2** on the migration of TNBC cells was assessed using a wound-healing assay. Briefly, cells were cultured in 6-well plates until confluency. Wounds were then made using a 10-μL pipette tip. After extensive washing with PBS, DMSO (control) or chromene compounds were added to the cells. The width of each wound was measured at the indicated time using an Evos XL inverted microscope at 40×. Wound closure, represented by the distance migrated (D) by cells for each wound was calculated as follows: D = width of the wound at t = 0 h − width of the wound at t = 4 or 10).

### 2.11. HUVEC Tube Formation Assay & Cell Viability

A 96-well plate was first coated with Matrigel matrix (Corning; 40–50 µL) then incubated for 1 h at 37 °C. Next, HUVECs (25,000 cells/well) were added to the coated plate along with DMSO (control) or **C1** or **C2** in duplicate. After 8 h, the tubes were photographed. The effect of **C1** or **C2** on the capacity of HUVECs to form capillary-like structures was assessed by measuring the length of newly formed tubules using Tube Formation Assay Image Analysis Solution software (WimTube, Wimasis (2016), Release 4.0, Onimagin Technologies SCA, Córdoba, Spain).

To assess the impact of **C1** or **C2** on HUVEC viability, ~9000 cells were seeded into 96-well plates and cultured for 24 h. The cells were then treated with **C1** or **C2** (or a negative control). Cell viability was measured at indicated time points using a Luminescent Cell Viability kit (Promega, Madison, WI, USA), as per the manufacturer’s protocol.

### 2.12. Tubulin Polymerization

MDA-MB-231 (2.8  ×  10^6^) cells were cultured in 100 mm culture dishes for 24 h before adding DMSO (control) or chromene compounds. The mixture was then left to culture for another 24 h. Control and treated cells were washed twice with pre-warmed PBS then lysed in hypotonic buffer (20 mM Tris HCl, pH 6.8, 1 mM MgCl 2, 5 mM amino caproic acid, 2 mM EGTA, 0.5% NP-40, 1 mM benzamidine, 2 mM PMSF, and 200 Units/mL aprotinin) at 37 °C for 5 min. The soluble (present in the supernatant) and polymerized tubulin (present in the pellet) fractions were separated by centrifugation at 15,000× *g* for 10 min at RT. The pellets were then suspended in lysis buffer and sonicated. The two fractions were then resolved by Western blotting analysis.

### 2.13. Molecular Docking

Three-dimensional (3D) protein structures of tubulin in complex with known inhibitors targeting distinct sites were obtained from the Protein Data Bank (PDB; https://www.rcsb.org). This included colchicine (PDB ID: 5NM5), vinblastine (PDB ID: 5J2T), laulimalide (PDB ID: 4O4H), and taxol (PDB ID: 6WVR). For each inhibitor, the inhibitor binding site harboring subunit was extracted, and its protein structure was pre-processed using Schrödinger Suite 2022-1 Protein Preparation Wizard (Schrödinger LLC, New York, NY, USA) using the default options. Hydrogen atoms were added, and the pH was set to 7 for the defining protonation states. Finally, the structures were optimized, and the energy was minimized to obtain a geometrically stable state. To facilitate molecular docking, a receptor grid was generated for each protein structure centered around the cocrystallized tubulin. In addition, the two-dimensional (2D) structure of chromenes **C1** and **C2** were drawn using Schrödinger Maestro and 2D Sketcher (Schrödinger LLC, New York, NY, USA). Ligand structures were then prepared for docking using Schrödinger Ligprep (Schrödinger LLC, New York, NY, USA). This step converts the 2D structure to a 3D structure, adds hydrogen atoms, generates various tautomers and ionization states, and optimizes the geometry of the molecules. Next, these structures were docked in the receptor grid of each of the proteins using Schrödinger Glide in extra precision (XP) mode [29]. The docked pose was then scored using the GlideScore scoring method [30]. We then analyzed the docked pose and visualized protein–ligand interactions using Schrödinger Maestro.

### 2.14. Statistical Analysis

Statistical analyses were conducted using SPSS version 21. Data were presented as mean  ±  SEM and were analyzed using one-way ANOVA. Statistical significance was defined as follows: * *p* < 0.05, ** *p* < 0.005, and *** *p* < 0.001.

## 3. Results

### 3.1. Synthesis of 2-Amino-3-Cyanochromene ***C1***

**C1** (Supplementary Figure S1) was synthesized using a three-component reaction comprising 1-naphthol, benzaldehyde, and malonitrile in ethanol in the presence of piperidine as a catalyst. Specific details of this reaction are included in the Supplementary Materials and Methods File S1. For the initial experiments, the mixture was refluxed in an oil bath at 80 °C for 6 h. However, we found that the reaction time could be significantly reduced to 5 min using microwave irradiation (dynamic power 25–30 W) and produced comparable yields (>90%). **C1** was purified by repeated recrystallization in hot ethanol. The observed structure showed good agreement between ^1^H NMR, ^13^C NMR, HRMS, and FT-IR datasets (Supplementary Figure S1a–d).

### 3.2. Synthesis of Schiff Base ***C2***

The Schiff base **C2** (Supplementary Figure S2) was prepared by heating a mixture of amine **C1** and salicylaldehyde in dry ethanol overnight. This procedure is described in detail in the Supplementary Materials and Methods. **C2** was produced when the resulting solution was allowed to cool at room temperature, filtered, and thoroughly washed with cold ethanol. ^1^H NMR (Supplementary Figure S2a), ^13^C NMR (Supplementary Figure S2b–d), and FT-IR (Supplementary Figure S2g) data indicated the absence of aldehyde groups in **C2**, and mass spectra indicated a mass peak at 402.1 Daltons (Supplementary Figure S2e,f) that corresponds to **C2**. Moreover, ^1^H NMR data indicated the presence of an imine = CH peak as a singlet at δ 9.49 ppm. The methine proton at C-4 (indicated by arrow in the structure of **C2**) appeared as two singlets (4.85 and 5.26 ppm), suggesting the presence of diastereoisomers in almost equal proportions. Moreover, ^13^C NMR spectra confirmed the presence of diastereoisomers. We did not attempt to separate them as **C2** readily decomposed in silica and alumina gels.

### 3.3. Chromenes ***C1*** and ***C2*** Selectively Inhibit the Cell Viability of TNBC Cells

**Figure 2:**
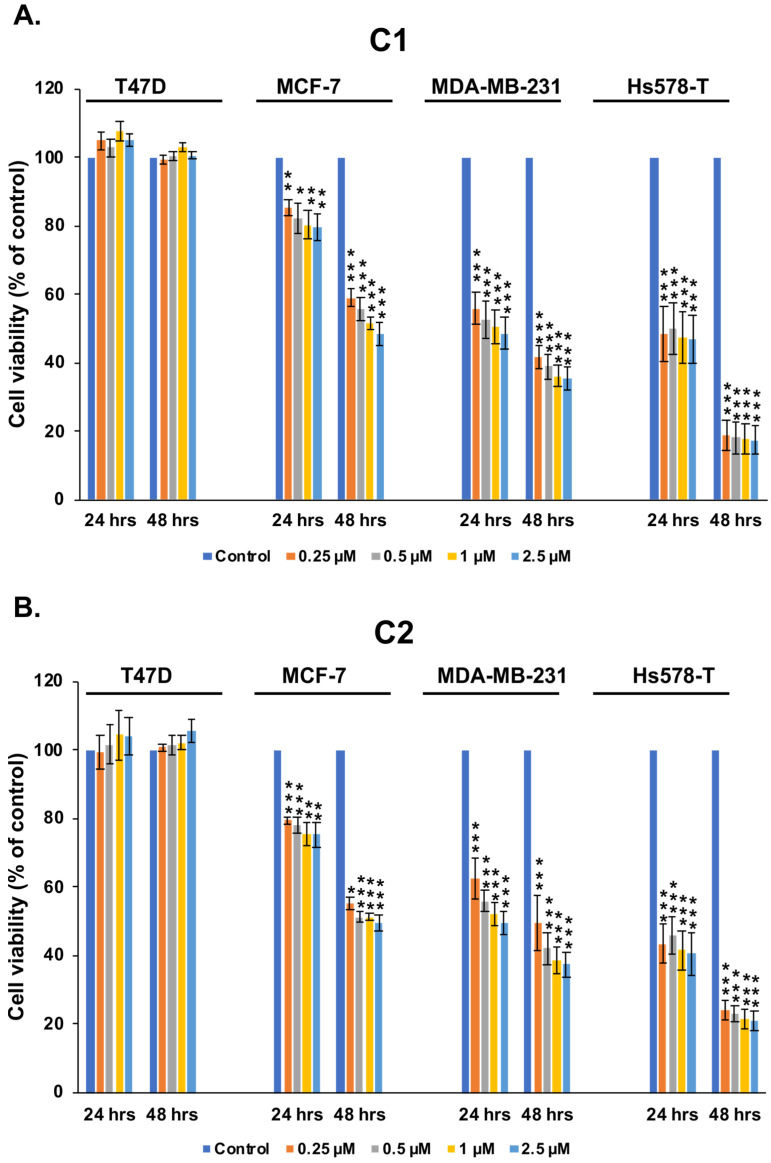
Chromenes **C1** and **C2** inhibit the cell viability of triple-negative breast cancer cells. The T47D, MCF-7, MDA-MB-231, and Hs578T cell lines after treatment with vehicle (0.1% DMSO) and the indicated concentrations of chromene **C1** (**A**) or **C2** (**B**) for 24 and 48 h. Cell viability was measured using the MTT assay as described in the text. All experiments were performed in three technical replicates and repeated at least four times. Columns and bars represent means and SEM, respectively. (*), (**), and (***) indicate that a condition was significantly different from the vehicle control at *p* < 0.05, *p* < 0.005, and *p* < 0.001, respectively.

We examined the effect of the newly synthesized chromenes **C1** and **C2** on the viability of two ER+, PR+, and HER2− luminal A breast cancer cell lines, MCF-7 and T47D. We also tested these chromenes with two TNBC cell lines: MDA-MB-231 and Hs578T. All cell lines were treated with **C1, C2**, or a negative control and examined using an MTT assay after treatment for 24 and 48 h. Interestingly, we found that the two TNBC lines MDA-MB-231 and Hs578T were more sensitive to **C1** (Figure 2A) and **C2** (Figure 2B) than MCF-7 (Figure 2A,B), which showed moderate sensitivity, or T47D (Figure 2A,B), which showed total resistance even at the highest concentration and after long exposure to both compounds. These data indicate that our newly modified chromenes selectively target TNBC cell lines.

**Figure 3:**
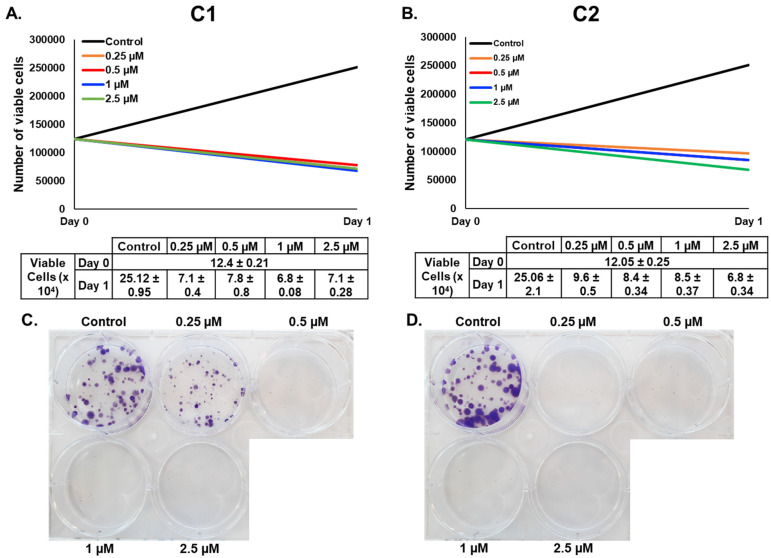
Chromenes **C1** and **C2** induce cell death and inhibit the anchorage-dependent, colony-forming ability of MDA-MB-231 triple-negative breast cancer cells. (A,B) Determination of cell viability via cell counting. MDA-MB-231 cells were treated with vehicle (0.1% DMSO) and the indicated concentrations of chromene **C1** (**A**) or **C2** (**B**) for 24 h. Cell viability was assessed using a Muse cell analyzer as described in the Materials and Methods. Data represent the mean  ±  SEM of three independent experiments. (**C**,**D**) Inhibition of MDA-MB-231 colony growth. MDA-MB-231 cells were cultured for 3 days before adding freshly prepared growth medium with or without various concentrations of chromene **C1** or **C2**.

The MTT assay measures metabolic activity and hence cannot distinguish between reduced metabolism due to cell stress or death. Therefore, we decided to further monitor the viability of **C1**- and **C2**-treated MDA-MB-231 and Hs578T cells using an assay that differentially stains dead and viable cells. As shown in Figure 3A,B, the number of untreated MDA-MBA-231 cells at day 1 increased relative to day 0, i.e., the day of treatment, whereas the number of **C1**- and **C2**-treated cells decreased compared with day 0. A similar result was also obtained for Hs578T cells (Supplementary Figure S3A,B). Taken together, our results indicate that **C1** and **C2** promote the death of TNBC cells.

To further confirm the potential of **C1** and **C2** against TNBC, we attempted to determine whether these two compounds affect the colony-forming ability of MDA-MB-231 and Hs578T cells. We found that even the lowest concentrations of **C1** and **C2** were able to markedly inhibit the colony growth of MDA-MB-231 (Figure 3C,D) and Hs578T cells (Supplementary Figure S3C,D).

### 3.4. ***C1*** and ***C2*** Induce Cell Death of TNBC Cells by Activating the Extrinsic Apoptotic Pathway

**Figure 4:**
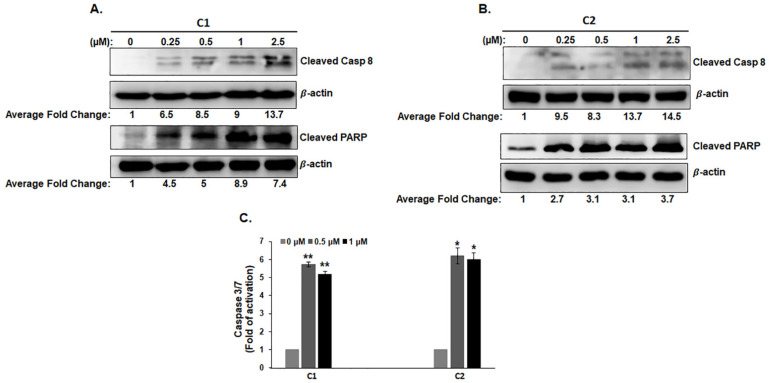
Chromenes **C1** and **C2** activate the extrinsic apoptotic pathway in MDA-MB-231 cells. (**A**,**B**) Western blotting analysis of caspase-8 and PARP cleavage in MDA-MB-231 cells treated with the indicated concentrations of **C1** (**A**) or **C2** (**B**). (**C**) The induction of caspase 3/7 in MDA-MB-231 cells after exposure to **C1** or **C2** for 24 h. Caspase 3/7 activity was normalized to the number of viable cells per well and was expressed as a fold difference in the degree of activation compared to control cells. Data represent the mean  ±  SEM of three independent experiments conducted in triplicate. (*) and (**) indicate statistically significantly different mean values at *p* < 0.05 and *p* < 0.005, respectively.

Next, we investigated whether the **C1**- and **C2**-mediated inhibition of cell viability of TNBC was mediated by activation of apoptosis by scoring markers of apoptosis. As shown in Figure 4 (for MDA-MB-231) and Supplementary Figure S4 (for Hs578T), treatment with chromenes **C1** and **C2** led to the accumulation of cleaved PARP and caspase 8 (an initiator caspase that is active in the extrinsic pathway) (Figure 4A,B), as well as the activation of caspase 3/7 (effector caspases) (Figure 4C). These data strongly suggest that **C1** and **C2** inhibit cell viability in TNBC cells, at least partially, by promoting cell death through the activation of the extrinsic apoptotic pathway.

### 3.5. ***C1*** and ***C2*** Induce Morphological Changes Associated with Massive Multinucleation and Accumulation of Double-Stranded DNA Breaks in TNBC Cells

**Figure 5:**
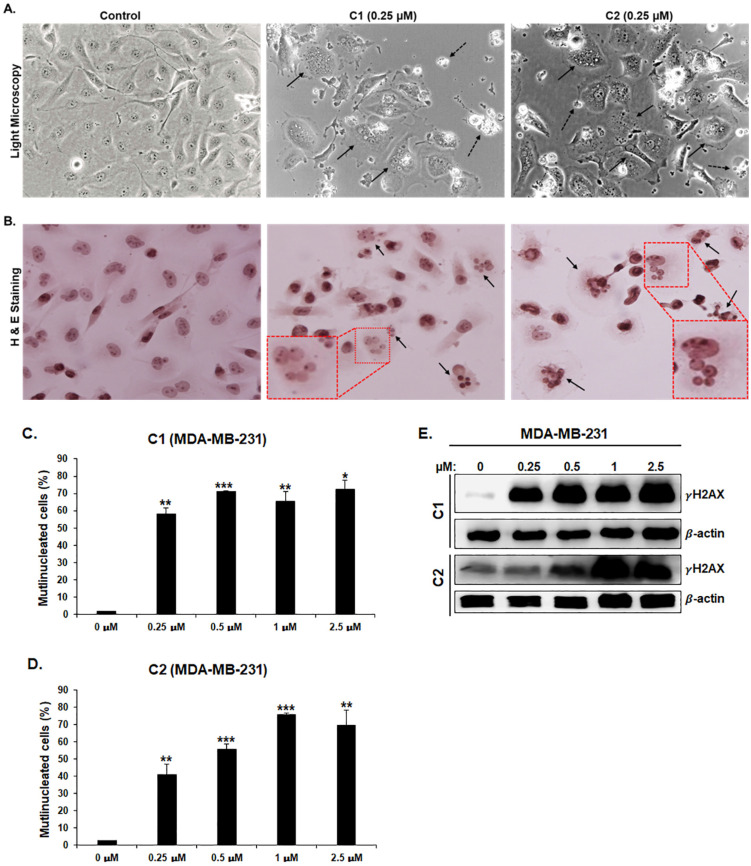
Chromenes **C1** and **C2** induce multinucleation in MDA-MB-231 cells. (**A**) Morphological changes observed in MDA-MB-231 cells treated for 24 h with or without 0.25 μM **C1** or **C2**. Cells were observed under an EVOS XL Core Cell Imaging System (Life Technologies, Carlsbad, CA, USA) at a magnification of 400×. (**B**) Treated MDA-MB-231 cells produced under the same conditions as those in section (**A**) were subjected to hematoxylin and eosin staining. Cells were photographed under an Olympus light microscope at 200× equipped with DP74. (**C**,**D**) Quantification of multinucleated cells treated with **C1** (**C**) or **C2** (**D**). (**E**) Upregulation of markers of double-strand DNA breaks (γH2AX) in MDA-MB-231 treated with **C1** or **C2**. MDA-MB-231 cells were then treated with or without the indicated concentrations of **C1** (**A**) or **C2** (**B**) for 24 h. Next, Western blotting was performed to assess the level of DNA damage by determining the level of γH2AX accumulation using anti-phospho-H2AX (ser 139) antibody. (*), (**) and (***) indicate statistically significantly different mean values at *p* < 0.05, *p* < 0.005 and *p* < 0.001, respectively.

Light microscopy observation of **C1**- and **C2**-treated MDA-MB-231 (Figure 5A) and Hs578T cells (Supplementary Figure S5A) revealed that the two chromenes induced several morphological changes. Some of the treated cells appeared small and rounded, which is characteristic of dying cells (Figure 4A, dashed arrows). Moreover, it is consistent with cell viability data. A more striking finding was the presence of a subpopulation of cells exhibiting a flattened and enlarged shape and, strikingly, a high level of multinucleation (Figure 5, plain arrow) compared with control cells (Figure 5A and Supplementary Figure S5A). The latter two are often associated with senescent cells. Multinucleation was further confirmed by examining chromene-treated MDA-MB-231 (Figure 5B) and Hs578T cells (Supplementary Figure S5B) using hematoxylin and eosin staining. We found several (2–6) nuclei of different sizes within the same cell. The frequency of chromene-induced multinucleation was 40–60% for **C1** and 40–70% for **C2** in MDA-MB-231 (Figure 5C,D), and 40–55% for both **C1** and **C2** in Hs578T (Supplementary Figure S5C,D).

It has also been reported that the accumulation of double-strand DNA breaks often occurs in cells undergoing prolonged mitotic delays. Similarly, multinucleated cells were also reported to display DNA damage [31]. We then sought to investigate whether **C1**- and **C2**-mediated effects were associated with DNA damage in MDA-MB-231 and Hs578T cells. The degree of DNA damage was assessed by measuring phosphorylated H2AX (γH2AX) levels in control and chromene-treated cells. Western blotting analysis revealed considerable γH2AX accumulation, a clear indication of DNA damage, in MDA-MB-231 (Figure 5E) and Hs578T (Supplementary Figure S5E).

### 3.6. ***C1*** and ***C2*** Induce Mitotic Arrest and Senescence in TNBC Cells

**Figure 6:**
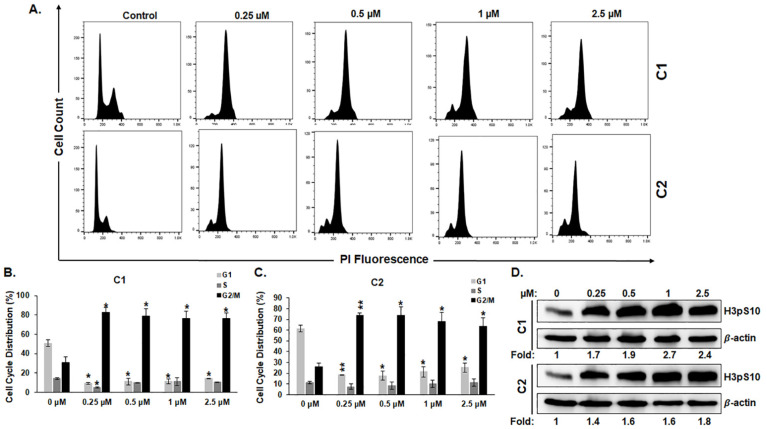
**C1** and **C2** induce a mitotic arrest in MDA-MB-231 cells. (**A**–**C**) Cell cycle distribution analysis showing MDA-MB-231 cells treated with and without **C1** or **C2** for 24 h. Values represent mean ± SEM of three independent experiments conducted in duplicate (* *p* < 0.05, ** *p* < 0.005). (**D**) Western blotting analysis of H3pSer10, a marker of the M phase, in MDA-MB-231 cells treated with or without **C1** or **C2**.

Cell multinucleation has been hypothesized to originate from failed mitosis [32]. This prompted us to examine the effect of **C1** and **C2** treatments on the progression of the cell cycle. To this effect, MDA-MB-231 and Hs578T cells were treated with and without the indicated concentrations of **C1** or **C2** for 24 h and were then subjected to cell cycle analysis. Treated cells exhibited a robust increase in the population of G2/M-arrested cells in both cell lines (Figure 6A–C and Supplementary Figure S5A–C). The number of G2/M-arrested cells increased for both compounds. These two cells represent up to 80% of the total cell population, a clear indication of massive cell cycle arrest. To determine if the cell cycle was specifically arrested at mitosis, we examined the phosphorylation of the marker of mitosis, namely histone H3 (H3pSer10) in cells treated with **C1** or **C2**. An increase in the level of H3pSer10, mediated by aurora kinase, implies mitosis. As shown in Figure 6D and Supplementary Figure S6D, both chromene compounds caused a significant increase in H3pSer021 levels in both cell lines. Taken together, these data confirm that chromenes **C1** and **C2** induce mitotic arrest in TNBC cells.

**Figure 7:**
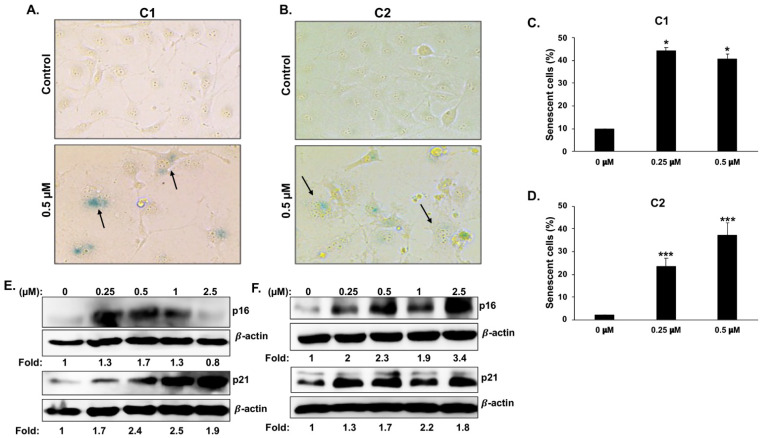
Senescence and upregulation of the cell cycle inhibitors p16 and p21 in **C1**- and **C2**-treated MDA-MB-231 cells. (**A**–**D**) Detection of senescence in **C1**- and **C2**-treated cells. MDA-MB-231 cells were incubated with or without chromene **C1** or **C2** for 48 h and were then stained for SA-β-galactosidase activity to detect senescence. Data are represented as mean ± SEM of three independent experiments (* p  <  0.05, *** p  <  0.001). (**E**,**F**) Upregulation of p16 and p21 protein levels. Cells were treated with the indicated concentrations of **C1** (**E**) or **C2** (**F**) for 24 h, after which the protein levels of p16 and p21 were determined through Western blotting analyses.

Next, we examined whether M-arrested chromene-treated cells also underwent senescence by looking at the expression of senescence-associated β-galactosidase (SA-β-Gal), a marker of senescence. We found that a significant number of cells treated with either **C1** or **C2** expressed SA-β-Gal (shown in blue) in MDA-MB-231 (Figure 7A–D) and Hs578T (Supplementary Figure S7A–D), respectively. Furthermore, we found that senescence occurred more frequently in multinucleated cells (Figure 7A,B, plain arrows). Taken together, our data demonstrate that chromenes **C1** and **C2** induce mitotic cell death and senescence in TNBC cells.

The involvement of p21 and p16, two cyclin-dependent kinase inhibitors (CKIs), in the induction and/or maintenance of senescence in response to anticancer therapy is well documented. This prompted us to ask whether senescence following administration of **C1** and **C2** is associated with the induction of p21 and/or p16. The results of typical Western blotting analyses, presented in Figure 7E,F, demonstrate a marked increase of both p16 and p21 proteins in **C1**- and **C2**-treated MDA-MB-231 cells. This result highlights the potential involvement of these two CKIs in **C1**- and **C2**-induced cell cycle arrest and senescence.

### 3.7. ***C1*** and ***C2*** Impair the Polymerization of Microtubules through Binding to the Colchicine Binding Site of β-Tubulin

**Figure 8:**
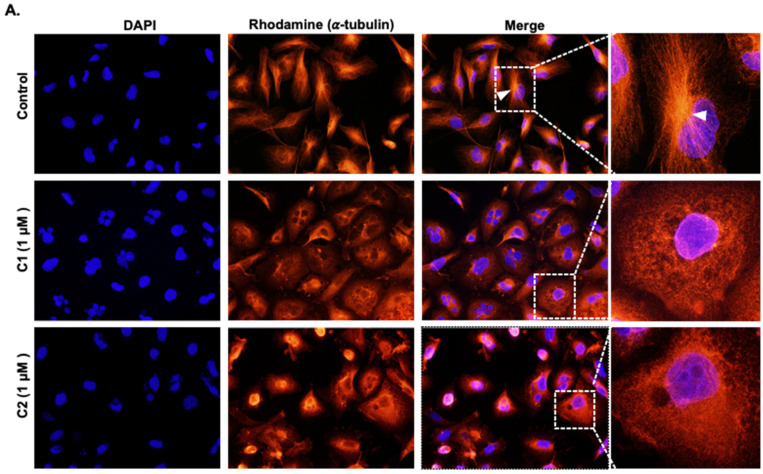

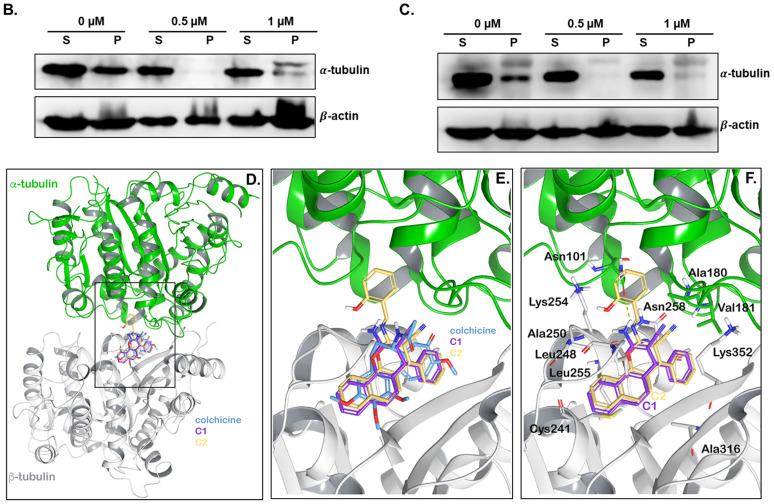
Chromenes **C1** and **C2** affect the integrity of cytoskeletal microtubule***s***. (**A**) Fluorescence microscopy analysis of MDA-MB-231 cells after 24 h treatment with 1 μM of **C1, C2,** or a negative control. Cells were stained with anti-α-tubulin, followed by an appropriate rhodamine-conjugated secondary antibody. The centrosome is indicated by a white arrowhead. (**B**,**C**) Chromene **C1** and **C2** inhibit microtubule polymerization in MDA-MB-231 cells. Cells were treated with or without **C1** or **C2** at concentrations of 0.5 and 1.0 μM for 24 h and lysed at 37 °C. Tubulin in polymers was separated from soluble tubulin by centrifugation as described in the Materials and Methods section. Equal amounts of each fraction of the supernatant (S) and the pellet (P) were analyzed by Western blotting for α-tubulin. (**D**–**F**) Docked pose of **C1** and **C2** in the colchicine binding site. (**D**,**E**) β-tubulin is presented in gray and α-tubulin in green. Docked **C1** is presented in violet, **C2** in yellow, and crystallized colchicine in blue. (**F**) A zoomed-in view of the boxed region in (**A**), with the key residues that **C1** and **C2** interact with.

Next, we sought to elucidate the mechanism through which **C1** and **C2** induce multinucleation in TNBC. Several microtubule-disrupting drugs are known to induce mitotic catastrophe associated with the accumulation of multinucleation in cancer cells. This prompted us to examine the integrity of the microtubule network in control and chromene-treated cells. MDA-MB-231 cells were treated with 1 μM of **C1, C2**, or a negative control for 24 h, after which they were fixed, stained with anti-α-tubulin antibody, and observed under a fluorescence microscope. In control cells, the microtubules concentrated in the perinuclear region and their extension toward the cytoplasm can be clearly seen (Figure 8A, upper panel). In contrast, no polymerized microtubule filaments were visible in **C1**- or **C2**-treated cells (Figure 8A, middle and lower panels). Instead, fluorescent α-tubulin dispersed throughout the cytoplasm, indicating a possible inhibition or defective polymerization of microtubules (Figure 8A, middle and lower panels).

To test whether our chromene compounds inhibited microtubule polymerization, we examined the relative amounts of different polymers. To this end, polymers and soluble forms were separated from control and **C1** or **C2**-treated MDA-MB-231 cells, and the amount of α-tubulin was resolved by Western blotting. As shown in Figure 8B,C, a large amount of αtubulin was present in a polymerized form in control and untreated cells. However, in cells treated with **C1** (Figure 8B) or **C2** (Figure 8C) at concentrations of 0.5 and 1 μM, the polymers were almost completely absent. Most of the tubulins were present in the soluble form. This result, along with immunofluorescence data, suggests that **C1** and **C2** act as microtubule-depolymerizing compounds.

We then conducted an in silico molecular docking study to test the potential affinity of **C1** and **C2** for α- and β-tubulin. Chromenes **C1** and **C2** were docked to four distinct inhibitor binding sites in α- and β-tubulin [33]—i.e., the colchicine, laulimalide, vinblastine, and taxol binding sites of tubulin. GlideScores analysis of the docked chromenes indicated very poor scores for the laulimalide, vinblastine, and taxol binding sites. **C1** and **C2** seemingly docked in the colchicine binding site, which is located in the interface between β-tubulin and α-tubulin. For this site, **C2** showed an XP GlideScore of −8.918 kcal/mol, whereas **C1** docked with an XP GlideScore of −8.299 kcal/mol. The core of both **C1** and **C2** adopted a similar pose in the colchicine binding site, which overlapped with how colchicine binds in this site (Figure 8D–F). Both **C1** and **C2** interacted with binding site residues primarily via hydrophobic interactions, as is evident in Supplementary Figure S8. The residues that contributed most to protein–ligand interactions were Lys352, Ala316, Asn258, Leu255, Lys254, Ala250, Leu248, and Cys241 of β-tubulin and to a lesser extent Val181, Ala180, and Asn101 of α-tubulin. The hydroxyl group of **C2** formed a hydrogen bond with Asn249 of β-tubulin, and the amino group of **C1** formed a hydrogen bond with Asn101 of α-tubulin.

### 3.8. ***C1*** and ***C2*** Cause Disruption of F-Actin Cytoskeleton Organization in TNBC

**Figure 9:**
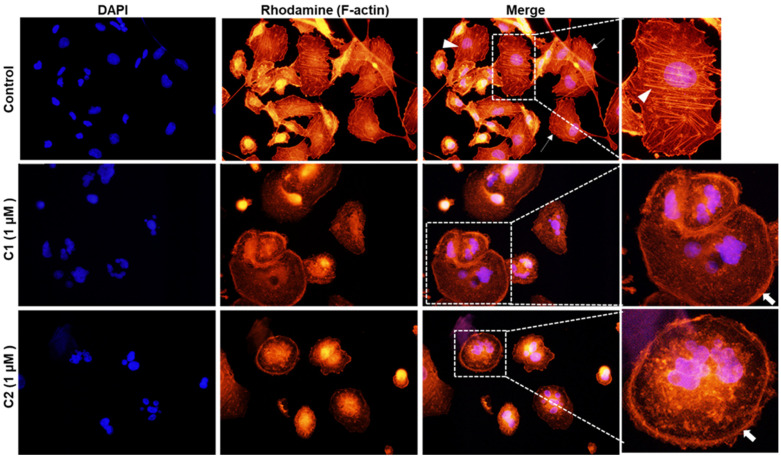
Defective polymerization of cytoskeletal actin filaments in **C1**- and **C2**-treated MDA-MB-231 cells. Fluorescence microscopy analysis of MDA-MB-231 cells treated with 1 μM of **C1** or **C2** for 24 h and stained with phalloidin.

Next, we proceeded to investigate the integrity of the actin cytoskeleton. To do this, we performed fluorescence microscopy to visualize the F-actin cytoskeleton in control and **C1**- and **C2**-treated MDA-MB-231 cells via phalloidin staining. As can be seen in Figure 9, DMSO-treated MDA-MB-231 cells exhibited the expected mesenchymal-like appearance with global stress fibers (arrowheads) and regional and occasional lamellipodia (thin dashed arrows). However, after treatment with **C1** or **C2**, patterns of complete abrogation of stress fibers were noted, and a dramatic formation of cortical actin fibers was observed (bold arrows). Moreover, treated cells were significantly larger in size than control cells. Such a major disruption in the localization of actin fibers as well as their assembly and disassembly, and the consequent aberrant cytokinesis, may explain why treated cells showed multinucleation. In addition, treated cells exhibited a more rounded morphology with no directional stress fibers, which is suggestive of migration suppression.

### 3.9. ***C1*** and ***C2*** Inhibit Cell Migration of MDA-MB-231 Cells

**Figure 10:**
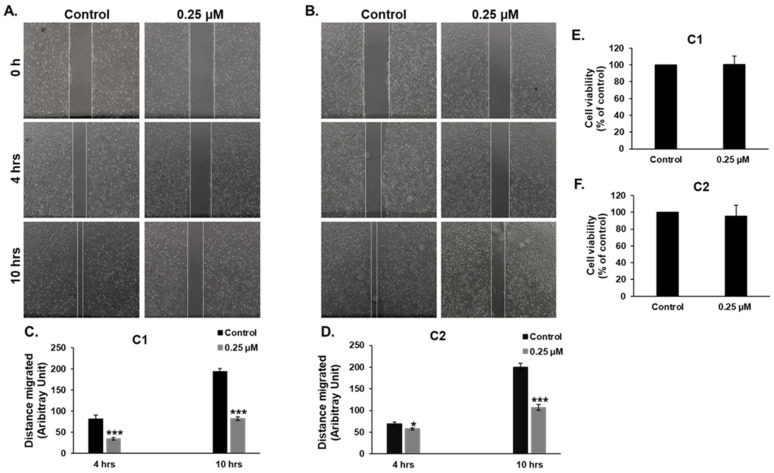
Chromenes **C1** and **C2** impair MDA-MB-231 cell motility. (**A**,**B**) Wounds were introduced in confluent monolayers of MDA-MB-231 cells before treating the cells with 0.25 μM of **C1** (**A**) or **C2** (**B**) or a negative control. The size of the wound was measured under a microscope (40× magnification) and was photographed using an EVOS XL Core Cell Imaging System (Life Technologies). (**C**,**D**) Quantification analysis of the wound-healing assay. Data are represented as mean ± SEM of the distance (in arbitrary units) migrated by cells after 4 and 10 h of treatment. Data are representative of three independent experiments. (* p  <  0.05, *** p  <  0.001). (**E**,**F**) Cell viability of MDA-MB-231 cells treated with **C1** (**E**) or **C2** (**F**) measured after 10 h treatment.

Cell migration and invasion are prerequisites for metastasis of TNBC from the primary tumor to distant organs. Moreover, it is well documented that the formation of fibrous strands of actin, known as F-actin, can promote cell division and migration, in addition to its role in the structural integrity of normal and cancer cells [34,35]. This prompted us to test the effect of the two chromene compounds on the migratory capacity of MDA-MB-231 and Hs578T. Our results showed that both **C1** and **C2** significantly inhibited the migration of highly migratory MDA-MB-231 cells (Figure 10A–D). To rule out the possibility that migration was inhibited due to the low number of cells, as a consequence of cell death, control and chromene-treated cells were collected from each well at the end of the experiment and the number of viable cells was counted. As is shown in Figure 10E,F, the number of viable cells did not decrease in chromene-treated wells compared to untreated wells.

### 3.10. Chromenes ***C1*** and ***C2*** Inhibit Angiogenesis

**Figure 11:**
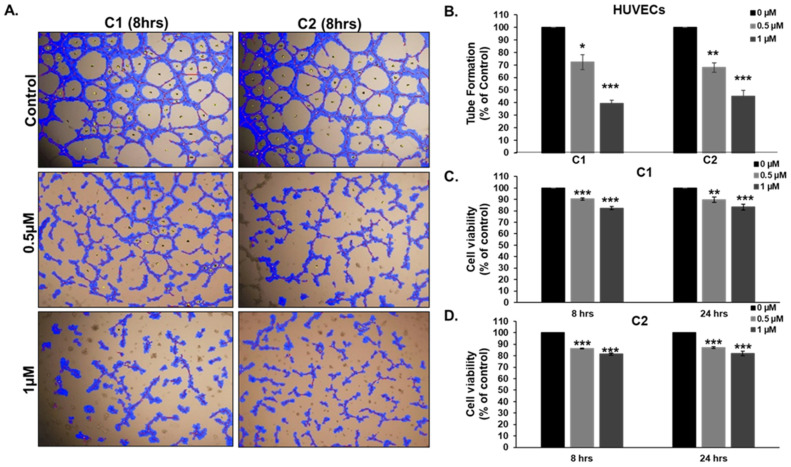
Inhibition of capillary-like structure formation by HUVECs in vitro. (**A**) Patterns of angiogenesis induced by human umbilical vein endothelial cells (HUVECs) cultured on matrigel matrix in 96-well plates with or without chromene **C1** or **C2**. (**B**) Quantification of tubule formation by control and chromene-treated HUVECs. Tube formation was evaluated by the length of tube-like structures containing connected cells. (**C**,**D**) Effect of **C1** or **C2** on the cell viability of HUVECs. The viability of HUVECs was measured 8 and 24 h post-treatment. Data represent mean  ±  SEM of three independent experiments each conducted in triplicate (* p  <  0.05, ** p  <  0.005, *** p  <  0.001).

Angiogenesis critically governs tumor growth and metastasis. Inhibiting this process can therefore inhibit tumor growth and spread. An intact cytoskeleton is critical for the various steps of angiogenesis and for the maintenance of newly formed structures [36]. We used HUVECs plated on matrigel-coated plates to examine the effect of **C1** and **C2** on the formation of capillary tubes in vitro. As shown in Figure 11A,B, treatment of HUVECs with either **C1** or **C2** compounds resulted in a significant reduction in tube formation in comparison to DMSO-treated cells after 8 h of treatment. The anti-angiogenic effect of **C1** or **C2** was observed in a concentration-dependent manner, with a 30% reduction observed at a concentration of 0.5 μM and a 60% reduction at a concentration of 1 μM. It is noteworthy that treatment with **C1** or **C2** caused only a small reduction in HUVECs’ viability after 8 h (~10%), and this effect did not further increase after 24 h of incubation with the chromene compounds (Figure 11C,D). Our results demonstrate the potent anti-angiogenic activity of the novel chromene compounds and suggest that the inhibition of tube formation occurs through disruption of mictotubules’ dynamics, and that reduction in cellular viability could account only for a very limited effect.

## 4. Discussion

TNBC constitutes a large proportion of breast cancer cases in young patients [37]. This subtype of breast cancer is one of the most aggressive forms, and is both characterized by a high mitotic index and often metastatic [38]. Moreover, almost all women with metastatic TNBC die from their disease. To date, and to the best of our knowledge, there is no targeted therapy yet approved for the treatment of TNBC, most probably because the heterogeneity of this disease makes it difficult to develop specific therapeutic treatments. At present, the standard therapeutic approach relies on cytotoxic chemotherapy. In this study, we synthesized two novel chromenes, **C1** and **C2**, that specifically target TNBC subtypes without a significant effect on hormone-responsive subtypes. Our chromenes induced mitotic arrest, cell multinucleation, and senescence. In addition, **C1** and **C2** both induced cell death via activation of the extrinsic apoptotic pathway. We demonstrated that **C1** and **C2** exert their anti-TNBC effects by binding to the colchicine binding site of β-tubulin, which leads to the destabilization of the microtubule network. Furthermore, we showed that **C1** and **C2** disrupt the organization of the actin filament network and inhibit cell migration. Finally, chromenes **C1** and **C2** display a potent antiangiogenic effect on HUVECs in vitro. Thus, our results indicate that these synthetic chromenes are potential potent new antitumor agents capable of specifically targeting TNBC subtypes.

Many chromenes (both natural and synthetic) have been reported to exert an antiproliferative activity against various cancer types. However, few have been reported as being microtubule-destabilizing agents [10]. Sp-6-27, a chromene derivative, was identified as a tubulin depolymerizing agent that exerted antiproliferative activity against cisplatin-sensitive (A2780) and -resistant (cis-A2780) ovarian cancer cell lines [21]. Sp-6-27 was shown to induce G2/M arrest and apoptotic cell death by activating the intrinsic pathway and, in addition, was able to inhibit the migration of A2780 cells. Crolibulin, a 4H-chromene currently in phase I/II of clinical trials with the National Cancer Institute, has been shown to be efficacious in treating a subset of patients with anaplastic thyroid cancer [39]. Crolibulin is described as an inhibitor of tubulin polymerization associated with G2/M arrest and apoptotic cell death. More recently, Pontes et al. showed that newly synthesized chromene derivatives (compounds **5c**, **5e**, and **5i**) reduced cell viability in luminal A, MCF-7, TNBC, and Hs578T breast cancer cell lines. However, Hs578T showed a much weaker response to these compounds than MCF-7 cells. Compound **5c**, the most effective compound, induced G2/M arrest and apoptotic cell death in MCF-7 cells. Morphological observation of MCF-7 treated with compound **5c** revealed disruption in the organization of the microtubule network [40]. Thus, the inhibition of the cell cycle at G2/M and activation of apoptosis is a common mechanism for microtubule-destabilizing chromenes. Our results are consistent with these findings. Here, we found that chromenes **C1** and **C2** induced cell mitotic arrest and cell death in TNBC. However, our compounds are the first chromenes that also show the induction of cell multinucleation, disruption of the F-actin cytoskeleton, and senescence. In addition, **C1** and **C2** are the first chromene compounds to exhibit selective activity against TNBC.

Microtubules and actin cooperate together to modify cell morphology and establish cell polarity. They do so either directly or by regulating signaling molecules [41]. Several signaling proteins, motor proteins, and other proteins associate, directly or indirectly, with microtubules and actin, and contribute to bridging the components of the cytoskeleton [42]. Thus, given the interconnection between various cytoskeletal polymers, changes in one polymer may affect other polymers. Studies have suggested that inhibition of microtubule polymerization by microtubule-destabilizing drugs, or their stabilization by microtubule-stabilizing compounds, can alter actin abundance. This can lead to changes in F-actin organization and loss of cell polarization, thus impacting the cytoskeletal network [43]. For example, Pletjushkina et al. reported that taxol induced a profound alteration in the distribution of actin filaments in rat fibroblasts [44]. A similar effect was also observed in paclitaxel- or docetaxel-treated MCF-7 cells [45]. Another study by Bijman et al. showed that docetaxel and vinblastine induced incorrect cell polarization and a reduction in stress fibers in HUVEC cells [46]. Our results were similar to these findings, in that we also observed the inhibition of microtubule polymerization by chromene administration, as well as a marked loss of actin stress fibers in MDA-MB-231 cells (Figure 10). Instead of stress fiber formation, we observed a marked increase of cortical actin fibers. To the best of our knowledge, our study is the first to show that chromenes, in addition to their microtubule destabilizing–activity, can also affect F-actin organization. It is notable that the mechanism by which microtubule disruption causes disruption of the organization of the actin cytoskeleton remains poorly explored.

Even though many chromene variants have been reported to inhibit tubulin polymerization, little is known regarding their mechanisms of action. Some studies have reported that chromene derivatives such as 4-aryl-4H-chromene (compound **1c**) [47] and 4H-chromene (compound **4b** and **4e**) [48] can inhibit tubulin polymerization by binding to the colchicine binding site. However, these studies relied only on in vitro microtubule polymerization assays and in silico docking data. No cellular or biochemical evidence supporting these conclusions has yet been provided. Here, we showed that our novel compounds **C1** and **C2** efficiently inhibited microtubule polymerization by immunofluorescence and Western blotting detection of microtubule filaments. In addition, our molecular docking data showed that **C1** and **C2** could dock best in the colchicine binding site, in a position similar to that of colchicine [49]. This docking was largely aided by hydrophobic interactions, which is characteristic of molecules that bind in this cavity [50]. The residues that interacted with colchicine were also observed to form hydrophobic contacts with both **C1** and **C2**. The binding of molecules in this site, in a colchicine-like pose, is expected to affect the ability of the α- and β-tubulin heterodimer to form a straight conformation.

When microtubule dynamics are affected, mitotic spindles cannot form properly. This will cause prolonged mitotic arrest, which in turn leads to growth arrest and cell death [51]. In some cases, cells can exit prolonged mitotic arrest, a phenomenon known as mitotic slippage, which leads to the formation of multiple nuclear compartments, commonly called multinucleation, as a result of improper chromosome segregation and cytokinesis [51,52]. Multinucleated cells can then enter either senescence, or simply die by apoptosis following slippage [52]. To date, the molecular mechanisms that determine the choice between cell death and senescence remain unclear. In some cancer cell lines, post-slippage apoptosis is the major type of cell death induced in response to anti-mitotic drug treatment [53]. In addition, DNA damage seems to play a major role in triggering post-slippage apoptosis [54]. Hart et al. showed that multinucleated cells exhibited the highest incidence of DNA damage, as revealed by the large number of γH2AX foci in over 90% of multinucleated cells [55]. Paclitaxel, an anti-mitotic drug that targets microtubule stabilization, was shown to induce post-slippage multinucleation associated with accumulation of DNA damage and massive apoptosis in Human Bone Osteosarcoma U-2 OS cells [54]. Moreover, the degree of post-slippage multinucleation is correlated with DNA damage and apoptosis [54]. Our results suggest that chromenes **C1** and **C2** target TNBC through a mechanism similar to that of paclitaxel. We also demonstrated that chromenes **C1** and **C2** induced mitotic arrest, multinucleation, and DNA damage, ultimately promoting apoptotic cell death.

Senescence has been reported to limit tumorigenesis and to foster anti-proliferative responses to cancer therapies [52]. In some cases, senescence is regarded as a tumor suppressing process, and prosenescence approaches have been proposed as promising cancer therapeutic frameworks [56]. Senescent cells, which are permanently growth-arrested but metabolically active, exhibit a complex senescent cell secretome, termed the senescence-associated secretory phenotype (SASP). SASP regulates the recruitment of immune cells to promote the immune clearance of senescent cells [57]. Growth arrest in senescent cells is achieved and maintained in part through the upregulation of the specific CKI p16, p21, and p27 [22,34,35,36]. p21 appears to be more involved in cell cycle blocks associated with early senescence, whereas p16 is more likely important for the maintenance of the senescence-associated phenotype [58]. Cells respond to DNA damage by inducing the expression of genes responsible for the senescence-associated phenotype [28]. The cellular choice between apoptosis or senescence is dependent, at least in part, on the level of DNA damage; minor DNA damage can induce senescence without causing cell death. Salinomycin, a monocarboxylic polyether antibiotic isolated from *Streptomyces albus* has been shown to induce both senescence and apoptosis in MDA-MB-231 cells, depending on the amount of DNA damage induced [28]. In that study, limited DNA damage triggered rapid senescence and extensive DNA damage triggered apoptosis [28]. Here, we showed that DNA damage occurred in multinucleated cells in response to chromene treatment. We hypothesized that the amount of DNA damage within each cell was the determining factor that determined whether multinucleated cells would undergo senescence or apoptosis.

Microtubules and actin filament function cooperatively to orchestrate cell motility, a process crucial for tumor invasion [59]. Actin stress fibers are important actors in cancer cell invasion—a hallmark of metastasis that remains the prime cause of cancer-related mortality—by mediating cellular polarity and motility [60]. Microtubule-targeting drugs (either microtubule stabilizing or destabilizing) have been reported to exert a potent inhibitory effect, at noncytotoxic concentrations, on the migration of various types of invasive cancer cells including TNBC cells [61]. However, very few studies have investigated the effect of microtubule-targeting drugs on the integrity of the actin cytoskeleton in cancer cells. For example, AB 186, a troglitazone derivative and microtubule-stabilizing compound, inhibits the migration of the two highly metastatic TNBC cell lines, MDA-MB-231 and Hs578T [62]. In addition, AB 186 has been shown to promote microtubule stabilization via direct binding to β-tubulin. AB 186 was also shown to affect an unorganized actin network in MDA-MB-231 cells [62]. Troglitazone itself was also shown to inhibit the migration of highly metastatic PK-1 (pancreatic) and ES-2 (ovarian) cancer cells via inhibition of actin polymerization, lamellipodia formation, and inducing a decrease in the number of actin stress fibers [63,64].

Remarkably, the inhibition of cell migration can also occur in cancer cells with defective actin organization independently of microtubule integrity. For example, Nimbolide, a major bioactive compound present in neem leaves, is an inhibitor that has been shown to induce the depolymerization of actin filaments. In one study, nimbolide dramatically and significantly inhibited the migration of the TNBC and MDA-MB-231 cell lines independently of microtubule network [65]. Here, we showed that chromenes **C1** and **C2** both significantly inhibited the formation of actin stress fibers and consequently impaired the migratory ability of MDA-MB-231 cells. Hence, our study provides support for the notion that the disruption of actin filaments, a consequence of microtubule polymerization or depolymerization, is sufficient to inhibit the migration of cancer cells. Therefore, this finding opens doors for further tests of the integrity of the F-actin network and its consequences in cancer cells treated with microtubule-targeting drugs. Moreover, the disruption or reorganization of the actin network may represent a common mechanism of action.

Microtubules play an essential role in orchestrating endothelial cell motility and angiogenesis. Angiogenesis, the process of new blood vessel formation, represents a keystone for both tumor progression and metastasis. Thus, targeting tumor vasculature can inhibit both tumor growth and metastasis [66], and is therefore regarded as a promising anticancer strategy. Microtubule-targeting drugs have gained much attention recently as potential tumor-selective antiangiogenic and vascular-disrupting agents as they possess the ability to suppress microtubule dynamics [67]. Furthermore, several microtubule-targeting drugs, including vinblastine [46] and colchicine [67], both of which destabilize microtubules, were shown to inhibit HUVEC motility, and thus angiogenesis, at concentrations that promote microtubule depolymerization but do not affect cell proliferation [46,67]. Similarly, subtoxic concentrations of paclitaxel [67], docetaxel [46], and epothilone [46], three microtubule-stabilizing drugs, have been reported to inhibit endothelial cell motility via interfering in microtubule dynamics [46,67]. Accordingly, suppressing microtubule dynamics, through microtubule destabilization or stabilization, may be sufficient to prevent angiogenesis. Here, we found that chromenes **C1** and **C2** both significantly prevented the formation of capillary-like structures in an in vitro tubule formation assay at concentrations that showed no cytotoxicity to HUVECs, even following long exposure times (e.g., 24 h). Consistent with previous findings, we postulate that chromenes **C1** and **C2** may inhibit angiogenesis, at least partly, via the destabilization of the microtubule network.

## 5. Conclusions

**Figure 12:**
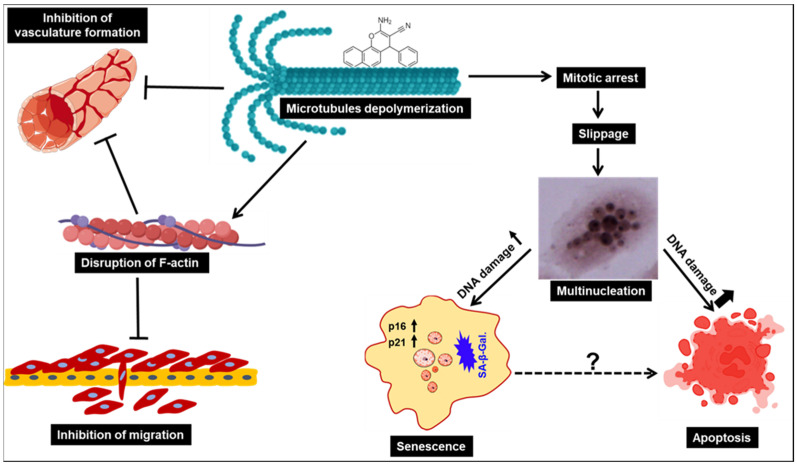
A hypothetical model for the anti-TNBC effect of chromenes **C1** and **C2**.

Our findings are summarized in Figure 12, which depicts the effect of two novel chromene derivatives C1 and C2, which we synthesized and characterized, against two TNBC cell lines. Treatment with C1 and C2 caused microtubule depolymerization that ultimately led to mitotic arrest in TNBC. These mitotically arrested cells then exited mitosis and gave rise to multinucleated cells, which in turn underwent either apoptosis, probably in response to extensive DNA damage, or remained permanently growth-arrested (senescent) via sustained expression of p21. Thus, C1 and C2, via disruption of the F-actin cytoskeleton, block the migration and thus invasiveness of TNBC cells. In addition, C1 and C2 act as potent antiangiogenic compounds, likely via inhibition of migration of HUVEC cells, due to microtubule depolymerization. Taken together, our results provide evidence for the potential of these compounds in breast cancer management, although further pre-clinical and clinical studies are still warranted.

## Data Availability

All data were provided in the present manuscript.

## Supplementary Materials

The following supporting information can be downloaded at: https://www.mdpi.com/article/10.3390/cancers15102682/s1, File S1: Supplementary method; File S2: Original blots; Figure S1: Synthesis and characterization of chromene compound **C1**. Figure S2: Synthesis and characterization of chromene compound **C2**. Figure S3: Chromene **C1** and **C2** induces cellular mortality and inhibit anchorage-dependent colony formation of Hs578T triple negative breast cancer cells. Figure S4: Chromene **C1** and **C2** activate the extrinsic apoptotic pathway in Hs578T cells; Figure S5: Chromene **C1** and **C2** induce multinucleation in Hs578T cells; Figure S6: **C1** and **C2** induce a mitotic arrest in Hs578T cells. Figure S7: Induction of senescence in **C1**- and **C2**-treated Hs578T cells. Figure S8: Ligand interaction diagram showing residues that interact with docked (A) **C1** and (B) **C2** and (C) co-crystallized colchicine.

## References

[1] Arnold M., Morgan E., Rumgay H., Mafra A., Singh D., Laversanne M., Vignat J., Gralow J.R., Cardoso F., Siesling S. (2022). Current and Future Burden of Breast Cancer: Global Statistics for 2020 and 2040. Breast.

[2] World Health Organization Breast Cancer. https://www.who.int/news-room/fact-sheets/detail/breast-cancer.

[3] Orrantia-Borunda E., Anchondo-Nuñez P., Acuña-Aguilar L.E., Gómez-Valles F.O., Ramírez-Valdespino C.A., Mayrovitz H.N. (2022). Subtypes of Breast Cancer. Breast Cancer.

[4] Yadav B.S., Chanana P., Jhamb S. (2015). Biomarkers in Triple Negative Breast Cancer: A Review. World J. Clin. Oncol..

[5] Geyer F.C., Pareja F., Weigelt B., Rakha E., Ellis I.O., Schnitt S.J., Reis-Filho J.S. (2017). The Spectrum of Triple-Negative Breast Disease: High- and Low-Grade Lesions. Am. J. Pathol..

[6] Gonçalves H., Guerra M.R., Duarte Cintra J.R., Fayer V.A., Brum I.V., Bustamante Teixeira M.T. (2018). Survival Study of Triple-Negative and Non-Triple-Negative Breast Cancer in a Brazilian Cohort. Clin. Med. Insights Oncol..

[7] Simões-Wüst A.P., Schürpf T., Hall J., Stahel R.A., Zangemeister-Wittke U. (2002). Bcl-2/Bcl-XL Bispecific Antisense Treatment Sensitizes Breast Carcinoma Cells to Doxorubicin, Paclitaxel and Cyclophosphamide. Breast Cancer Res. Treat..

[8] Inao T., Iida Y., Moritani T., Okimoto T., Tanino R., Kotani H., Harada M. (2018). Bcl-2 Inhibition Sensitizes Triple-Negative Human Breast Cancer Cells to Doxorubicin. Oncotarget.

[9] Nurgali K., Jagoe R.T., Abalo R. (2018). Editorial: Adverse Effects of Cancer Chemotherapy: Anything New to Improve Tolerance and Reduce Sequelae?. Front. Pharmacol..

[10] Raj V., Lee J. (2020). 2H/4H-Chromenes-A Versatile Biologically Attractive Scaffold. Front. Chem..

[11] Thomas N., Zachariah S. (2013). Pharmacological Activities of Chromene Derivatives: An Overview. Asian J. Pharm. Clin. Res..

[12] Katiyar M.K., Dhakad G.K., Shivani, Arora S., Bhagat S., Arora T., Kumar R. (2022). Synthetic Strategies and Pharmacological Activities of Chromene and Its Derivatives: An Overview. J. Mol. Struct..

[13] Li J., Wang X.-L., Fang Y.-C., Wang C.-Y. (2010). Tephrosin-Induced Autophagic Cell Death in A549 Non-Small Cell Lung Cancer Cells. J. Asian Nat. Prod. Res..

[14] Du J., Jiang F., Xu S.-S., Huang Z.-F., Chen L.-L., Li L. (2021). Tephrosin Induces Apoptosis of Human Pancreatic Cancer Cells through the Generation of Reactive Oxygen Species. J. Cancer.

[15] Ekowati H., Astuti I., Mustofa M. (2010). Anticancer Activity Of Calanone On Hela Cell Line. Indones. J. Chem..

[16] Guilbaud N., Kraus-Berthier L., Meyer-Losic F., Malivet V., Chacun C., Jan M., Tillequin F., Michel S., Koch M., Pfeiffer B. (2001). Marked Antitumor Activity of a New Potent Acronycine Derivative in Orthotopic Models of Human Solid Tumors. Clin. Cancer Res..

[17] Nishino H., Okuyama T., Takata M., Shibata S., Tokuda H., Takayasu J., Hasegawa T., Nishino A., Ueyama H., Iwashima A. (1990). Studies on the Anti-Tumor-Promoting Activity of Naturally Occurring Substances. IV. Pd-II [(+)Anomalin, (+)Praeruptorin B], a Seselin-Type Coumarin, Inhibits the Promotion of Skin Tumor Formation by 12-O-Tetradecanoylphorbol-13-Acetate in 7,12-Dimethylbenz[a]Anthracene-Initiated Mice. Carcinogenesis.

[18] Lima C.F., Costa M., Proença M.F., Pereira-Wilson C. (2015). Novel Structurally Similar Chromene Derivatives with Opposing Effects on P53 and Apoptosis Mechanisms in Colorectal HCT116 Cancer Cells. Eur. J. Pharm. Sci..

[19] Asgari F., Mahinpour R., Moradi L., Haghighipour N. (2020). The Chromene Derivative 4-Clpgc Inhibits Cell Proliferation and Induces Apoptosis in the K562 Cell Line. J. Cell Commun. Signal..

[20] Zhang W.-H., Chen S., Liu X.-L., Bing-Lin N., Liu X.-W., Zhou Y. (2020). Study on Antitumor Activities of the Chrysin-Chromene-Spirooxindole on Lewis Lung Carcinoma C57BL/6 Mice in vivo. Bioorg. Med. Chem. Lett..

[21] Kulshrestha A., Katara G.K., Ibrahim S.A., Patil R., Patil S.A., Beaman K.D. (2017). Microtubule Inhibitor, SP-6-27 Inhibits Angiogenesis and Induces Apoptosis in Ovarian Cancer Cells. Oncotarget.

[22] Puppala M., Zhao X., Casemore D., Zhou B., Aridoss G., Narayanapillai S., Xing C. (2016). 4H-Chromene-Based Anticancer Agents towards Multi-Drug Resistant HL60/MX2 Human Leukemia: SAR at the 4th and 6th Positions. Bioorg. Med. Chem..

[23] Kulshrestha A., Ibrahim S.A., Katara G.K., Patil R., Patil S., Beaman K.D. (2016). Abstract 1231: Novel Chromene Analogs as Small-Molecule Microtubule Destabilizers for the Treatment of Chemo-Resistant Ovarian Cancer. Cancer Res..

[24] Patil S.A., Patil R., Pfeffer L.M., Miller D.D. (2013). Chromenes: Potential New Chemotherapeutic Agents for Cancer. Future Med. Chem..

[25] Cai S.X., Drewe J., Kemnitzer W. (2009). Discovery of 4-Aryl-4H-Chromenes as Potent Apoptosis Inducers Using a Cell- and Caspase-Based Anti-Cancer Screening Apoptosis Program (ASAP): SAR Studies and the Identification of Novel Vascular Disrupting Agents. Anticancer Agents Med. Chem..

[26] Fallah-Tafti A., Tiwari R., Shirazi A.N., Akbarzadeh T., Mandal D., Shafiee A., Parang K., Foroumadi A. (2011). 4-Aryl-4H-Chromene-3-Carbonitrile Derivatives: Evaluation of Src Kinase Inhibitory and Anticancer Activities. Med. Chem..

[27] Benhalilou N., Alsamri H., Alneyadi A., Athamneh K., Alrashedi A., Altamimi N., Al Dhaheri Y., Eid A.H., Iratni R. (2019). Origanum Majorana Ethanolic Extract Promotes Colorectal Cancer Cell Death by Triggering Abortive Autophagy and Activation of the Extrinsic Apoptotic Pathway. Front. Oncol..

[28] Al Dhaheri Y., Attoub S., Arafat K., Abuqamar S., Eid A., Al Faresi N., Iratni R. (2013). Salinomycin Induces Apoptosis and Senescence in Breast Cancer: Upregulation of P21, Downregulation of Survivin and Histone H3 and H4 Hyperacetylation. Biochim. Biophys. Acta.

[29] Friesner R.A., Murphy R.B., Repasky M.P., Frye L.L., Greenwood J.R., Halgren T.A., Sanschagrin P.C., Mainz D.T. (2006). Extra Precision Glide: Docking and Scoring Incorporating a Model of Hydrophobic Enclosure for Protein-Ligand Complexes. J. Med. Chem..

[30] Friesner R.A., Banks J.L., Murphy R.B., Halgren T.A., Klicic J.J., Mainz D.T., Repasky M.P., Knoll E.H., Shelley M., Perry J.K. (2004). Glide: A New Approach for Rapid, Accurate Docking and Scoring. 1. Method and Assessment of Docking Accuracy. J. Med. Chem..

[31] Hayashi M.T., Cesare A.J., Fitzpatrick J.A.J., Lazzerini-Denchi E., Karlseder J. (2012). A Telomere-Dependent DNA Damage Checkpoint Induced by Prolonged Mitotic Arrest. Nat. Struct. Mol. Biol..

[32] Dikovskaya D., Cole J.J., Mason S.M., Nixon C., Karim S.A., McGarry L., Clark W., Hewitt R.N., Sammons M.A., Zhu J. (2015). Mitotic Stress Is an Integral Part of the Oncogene-Induced Senescence Program That Promotes Multinucleation and Cell Cycle Arrest. Cell Rep..

[33] Lu Y., Chen J., Xiao M., Li W., Miller D.D. (2012). An Overview of Tubulin Inhibitors That Interact with the Colchicine Binding Site. Pharm. Res..

[34] Dominguez R., Holmes K.C. (2011). Actin Structure and Function. Annu. Rev. Biophys..

[35] Perrin B.J., Ervasti J.M. (2010). The Actin Gene Family: Function Follows Isoform. Cytoskeleton.

[36] Bayless K.J., Johnson G.A. (2011). Role of the Cytoskeleton in Formation and Maintenance of Angiogenic Sprouts. J. Vasc. Res..

[37] Jenkins E.O., Deal A.M., Anders C.K., Prat A., Perou C.M., Carey L.A., Muss H.B. (2014). Age-Specific Changes in Intrinsic Breast Cancer Subtypes: A Focus on Older Women. Oncologist.

[38] Tzikas A.-K., Nemes S., Linderholm B.K. (2020). A Comparison between Young and Old Patients with Triple-Negative Breast Cancer: Biology, Survival and Metastatic Patterns. Breast Cancer Res. Treat..

[39] Gulley J. (2017). A Phase I/II Trial of Crolibulin (EPC2407) Plus Cisplatin in Adults with Solid Tumors with a Focus on Anaplastic Thyroid Cancer (ATC). https://clinicaltrials.gov/.

[40] Pontes O., Costa M., Santos F., Sampaio-Marques B., Dias T., Ludovico P., Baltazar F., Proença F. (2018). Exploitation of New Chalcones and 4H-Chromenes as Agents for Cancer Treatment. Eur. J. Med. Chem..

[41] Etienne-Manneville S. (2004). Actin and Microtubules in Cell Motility: Which One Is in Control?. Traffic.

[42] Mohan R., John A. (2015). Microtubule-Associated Proteins as Direct Crosslinkers of Actin Filaments and Microtubules. IUBMB Life.

[43] Bates D., Eastman A. (2017). Microtubule Destabilising Agents: Far More than Just Antimitotic Anticancer Drugs. Br. J. Clin. Pharmacol..

[44] Pletjushkina O.J., Ivanova O.J., Kaverina I.N., Vasiliev J.M. (1994). Taxol-Treated Fibroblasts Acquire an Epithelioid Shape and a Circular Pattern of Actin Bundles. Exp. Cell Res..

[45] Rosenblum M.D., Shivers R.R. (2000). “Rings” of F-Actin Form around the Nucleus in Cultured Human MCF7 Adenocarcinoma Cells upon Exposure to Both Taxol and Taxotere. Comp. Biochem. Physiol. Part C Pharmacol. Toxicol. Endocrinol..

[46] Bijman M.N.A., van Nieuw Amerongen G.P., Laurens N., van Hinsbergh V.W.M., Boven E. (2006). Microtubule-Targeting Agents Inhibit Angiogenesis at Subtoxic Concentrations, a Process Associated with Inhibition of Rac1 and Cdc42 Activity and Changes in the Endothelial Cytoskeleton. Mol. Cancer Ther..

[47] Kemnitzer W., Drewe J., Jiang S., Zhang H., Wang Y., Zhao J., Jia S., Herich J., Labreque D., Storer R. (2004). Discovery of 4-Aryl-4H-Chromenes as a New Series of Apoptosis Inducers Using a Cell- and Caspase-Based High-Throughput Screening Assay. 1. Structure-Activity Relationships of the 4-Aryl Group. J. Med. Chem..

[48] Patil S.A., Wang J., Li X.S., Chen J., Jones T.S., Hosni-Ahmed A., Patil R., Seibel W.L., Li W., Miller D.D. (2012). New Substituted 4H-Chromenes as Anticancer Agents. Bioorg. Med. Chem. Lett..

[49] Weinert T., Olieric N., Cheng R., Brünle S., James D., Ozerov D., Gashi D., Vera L., Marsh M., Jaeger K. (2017). Serial Millisecond Crystallography for Routine Room-Temperature Structure Determination at Synchrotrons. Nat. Commun..

[50] Wang J., Miller D.D., Li W. (2022). Molecular Interactions at the Colchicine Binding Site in Tubulin: An X-ray Crystallography Perspective. Drug Discov. Today.

[51] Di Rorà A.G.L., Martinelli G., Simonetti G. (2019). The Balance between Mitotic Death and Mitotic Slippage in Acute Leukemia: A New Therapeutic Window?. J. Hematol. Oncol..

[52] Cheng B., Crasta K. (2017). Consequences of Mitotic Slippage for Antimicrotubule Drug Therapy. Endocr. Relat. Cancer.

[53] Shi J., Orth J.D., Mitchison T. (2008). Cell Type Variation in Responses to Antimitotic Drugs That Target Microtubules and Kinesin-5. Cancer Res..

[54] Zhu Y., Zhou Y., Shi J. (2014). Post-Slippage Multinucleation Renders Cytotoxic Variation in Anti-Mitotic Drugs That Target the Microtubules or Mitotic Spindle. Cell Cycle.

[55] Hart M., Adams S.D., Draviam V.M. (2021). Multinucleation Associated DNA Damage Blocks Proliferation in P53-Compromised Cells. Commun. Biol..

[56] You J., Dong R., Ying M., He Q., Cao J., Yang B. (2019). Cellular Senescence and Anti-Cancer Therapy. Curr. Drug Targets.

[57] Prata L.G.P.L., Ovsyannikova I.G., Tchkonia T., Kirkland J.L. (2018). Senescent Cell Clearance by the Immune System: Emerging Therapeutic Opportunities. Semin. Immunol..

[58] Stein G.H., Drullinger L.F., Soulard A., Dulić V. (1999). Differential Roles for Cyclin-Dependent Kinase Inhibitors P21 and P16 in the Mechanisms of Senescence and Differentiation in Human Fibroblasts. Mol. Cell Biol..

[59] Aseervatham J. (2020). Cytoskeletal Remodeling in Cancer. Biology.

[60] Fischer R.S., Sun X., Baird M.A., Hourwitz M.J., Seo B.R., Pasapera A.M., Mehta S.B., Losert W., Fischbach C., Fourkas J.T. (2021). Contractility, Focal Adhesion Orientation, and Stress Fiber Orientation Drive Cancer Cell Polarity and Migration along Wavy ECM Substrates. Proc. Natl. Acad. Sci. USA.

[61] Čermák V., Dostál V., Jelínek M., Libusová L., Kovář J., Rösel D., Brábek J. (2020). Microtubule-Targeting Agents and Their Impact on Cancer Treatment. Eur. J. Cell Biol..

[62] Geoffroy M., Lemesle M., Kleinclauss A., Mazerbourg S., Batista L., Barberi-Heyob M., Bastogne T., Boireau W., Rouleau A., Dupommier D. (2022). AB186 Inhibits Migration of Triple-Negative Breast Cancer Cells and Interacts with α-Tubulin. Int. J. Mol. Sci..

[63] Wang P.-S., Chou F.-S., Porchia L., Saji M., Pinzone J.J. (2008). Troglitazone Inhibits Cell Migration, Adhesion, and Spreading by Modulating Cytoskeletal Rearrangement in Human Breast Cancer Cells. Mol. Carcinog..

[64] Yang Y.-C., Ho T.-C., Chen S.-L., Lai H.-Y., Wu J.-Y., Tsao Y.-P. (2007). Inhibition of Cell Motility by Troglitazone in Human Ovarian Carcinoma Cell Line. BMC Cancer.

[65] Arumugam A., Subramani R., Lakshmanaswamy R. (2021). Involvement of Actin Cytoskeletal Modifications in the Inhibition of Triple-Negative Breast Cancer Growth and Metastasis by Nimbolide. Mol. Ther. Oncolytics.

[66] Pasquier E., André N., Braguer D. (2007). Targeting Microtubules to Inhibit Angiogenesis and Disrupt Tumour Vasculature: Implications for Cancer Treatment. Curr. Cancer Drug Targets.

[67] Ganguly A., Yang H., Zhang H., Cabral F., Patel K.D. (2013). Microtubule Dynamics Control Tail Retraction in Migrating Vascular Endothelial Cells. Mol. Cancer Ther..

