# Supplementary material for: Synthesis of New Chromene Derivatives Targeting Triple-Negative Breast Cancer Cells
Source: Cancers (Basel). 2023 May 9;15(10):2682. doi: 10.3390/cancers15102682 (PMC10216081; doi:10.3390/cancers15102682)
Supplement: Supplementary file 1 [file cancers-15-02682-s001.zip › cancers-2286253-File S2 - Uncropped Western Blot.pdf]

# Uncropped Western blot
# Main Body text

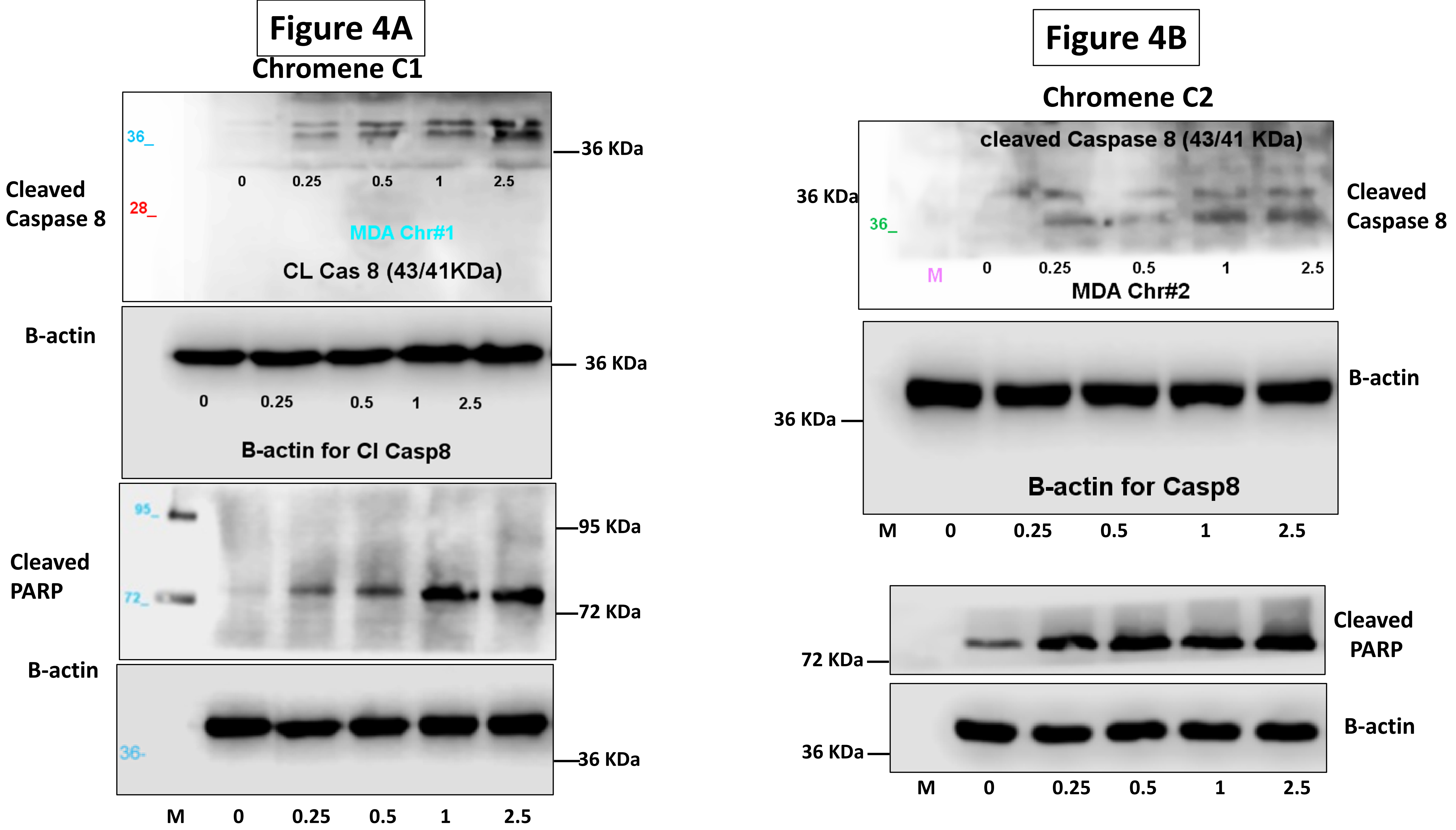
Figure 4A
Chromene C1
Cleaved Caspase 8
36_
28_
0
0.25
0.5
1
2.5
MDA Chr#1
CL Cas 8 (43/41KDa)
36 KDa
B-actin
0
0.25
0.5
1
2.5
B-actin for Cl Casp8
36 KDa
Cleaved PARP
95_
72_
95 KDa
72 KDa
B-actin
36-
36 KDa
M
0
0.25
0.5
1
2.5
Figure 4B
Chromene C2
cleaved Caspase 8 (43/41 KDa)
36 KDa
36_
M
0
0.25
0.5
1
2.5
MDA Chr#2
Cleaved Caspase 8
B-actin
36 KDa
B-actin for Casp8
M
0
0.25
0.5
1
2.5
Cleaved PARP
72 KDa
B-actin
36 KDa
M
0
0.25
0.5
1
2.5

# Figure 5E

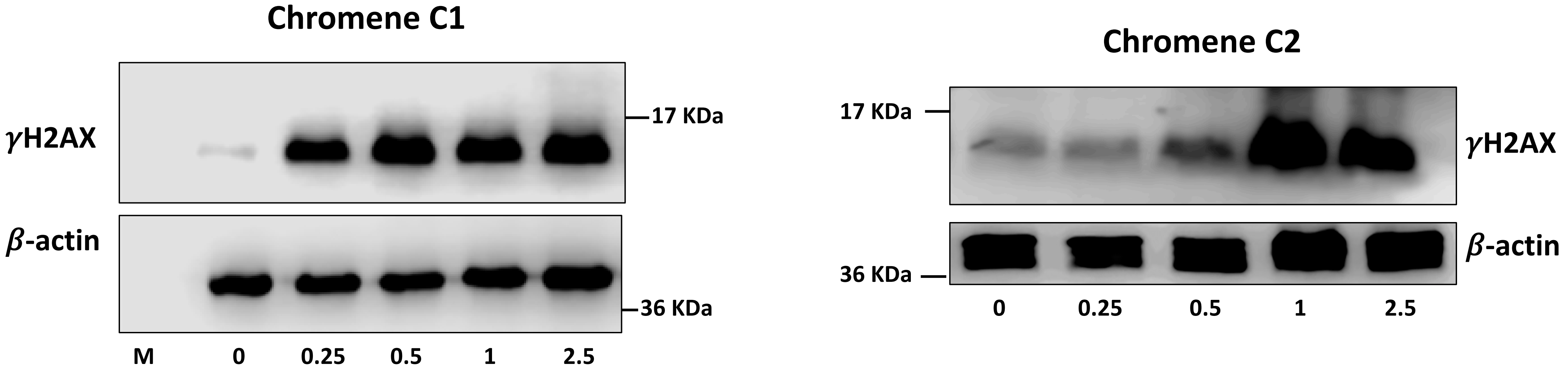

Chromene C1
γH2AX
β-actin
17 KDa
36 KDa
M
0
0.25
0.5
1
2.5
Chromene C2
17 KDa
36 KDa
γH2AX
β-actin
0
0.25
0.5
1
2.5

**Figure 6D**

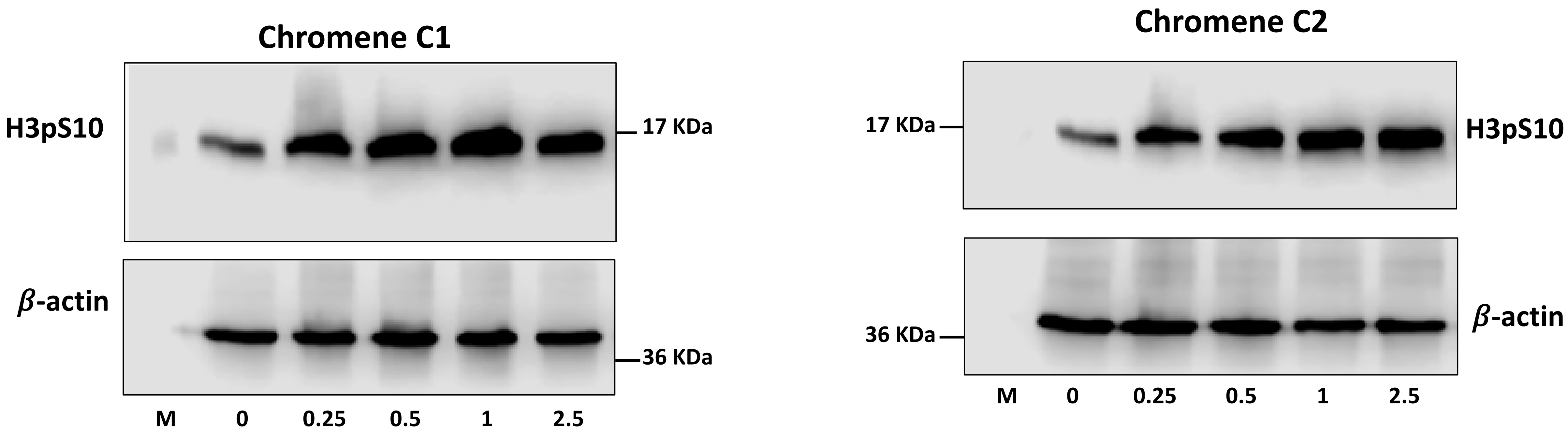
Chromene C1
H3pS10
17 KDa
β-actin
36 KDa
M
0
0.25
0.5
1
2.5
Chromene C2
17 KDa
H3pS10
36 KDa
β-actin
M
0
0.25
0.5
1
2.5

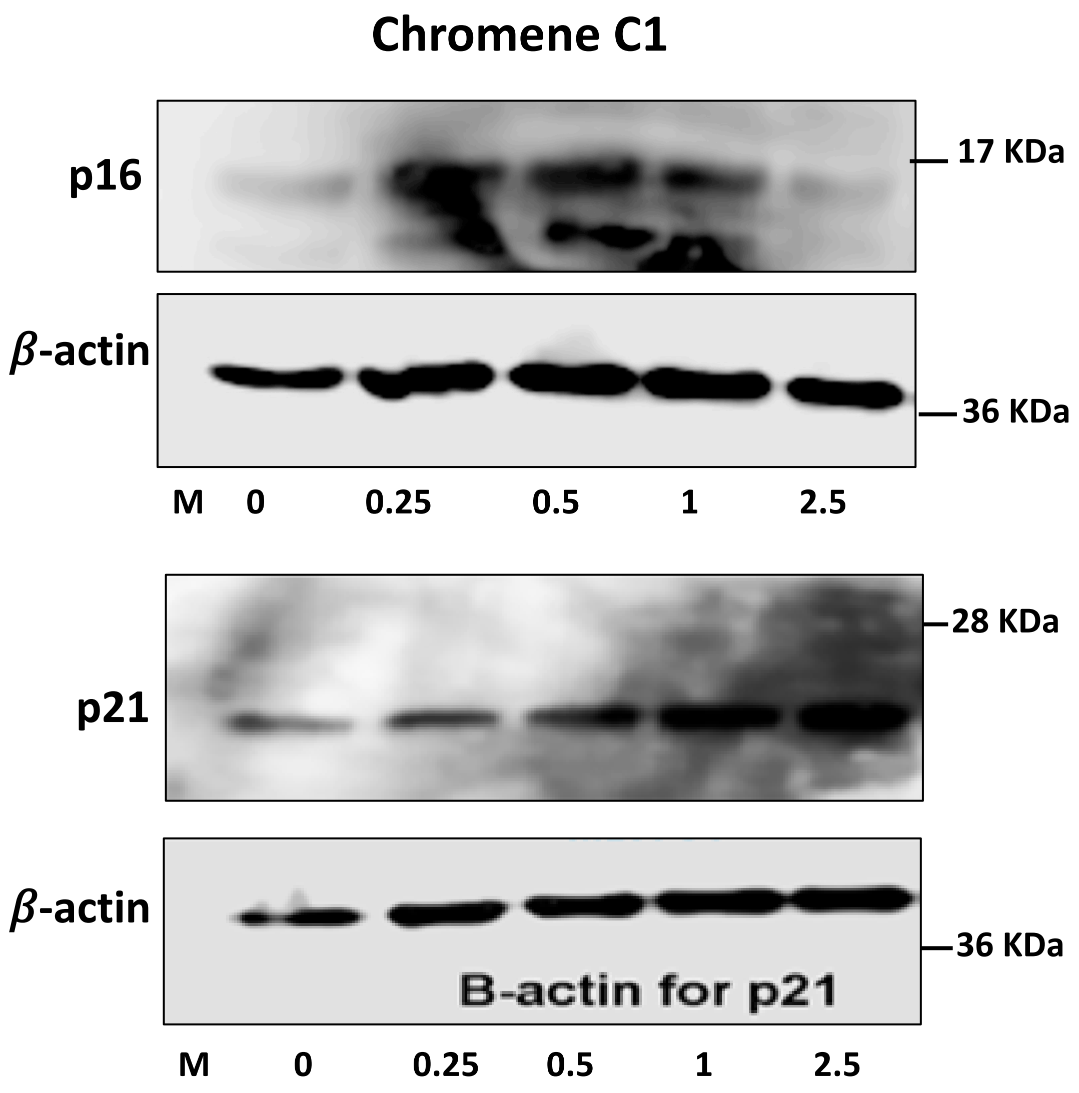


Figure 7E

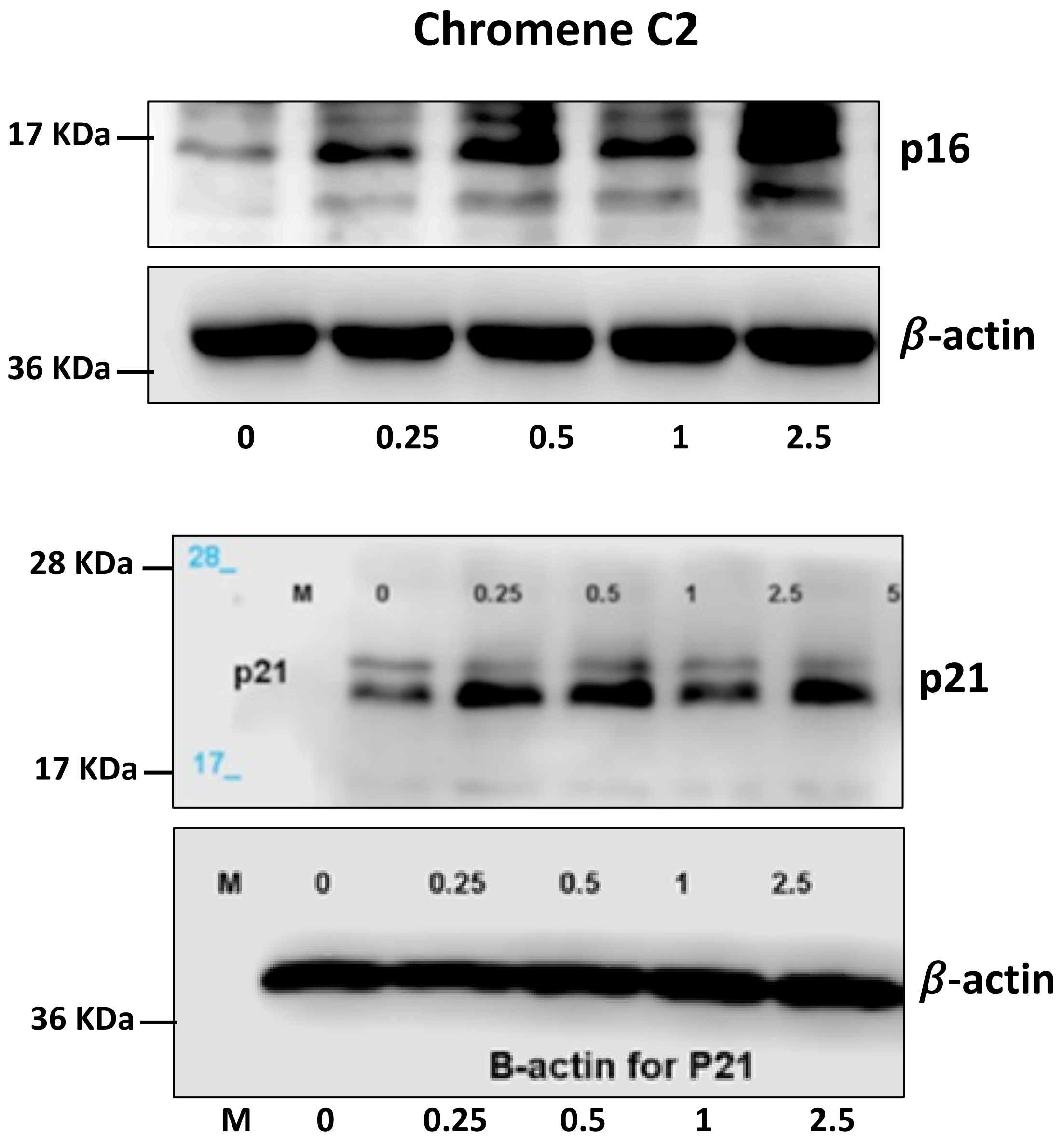


Figure 7F

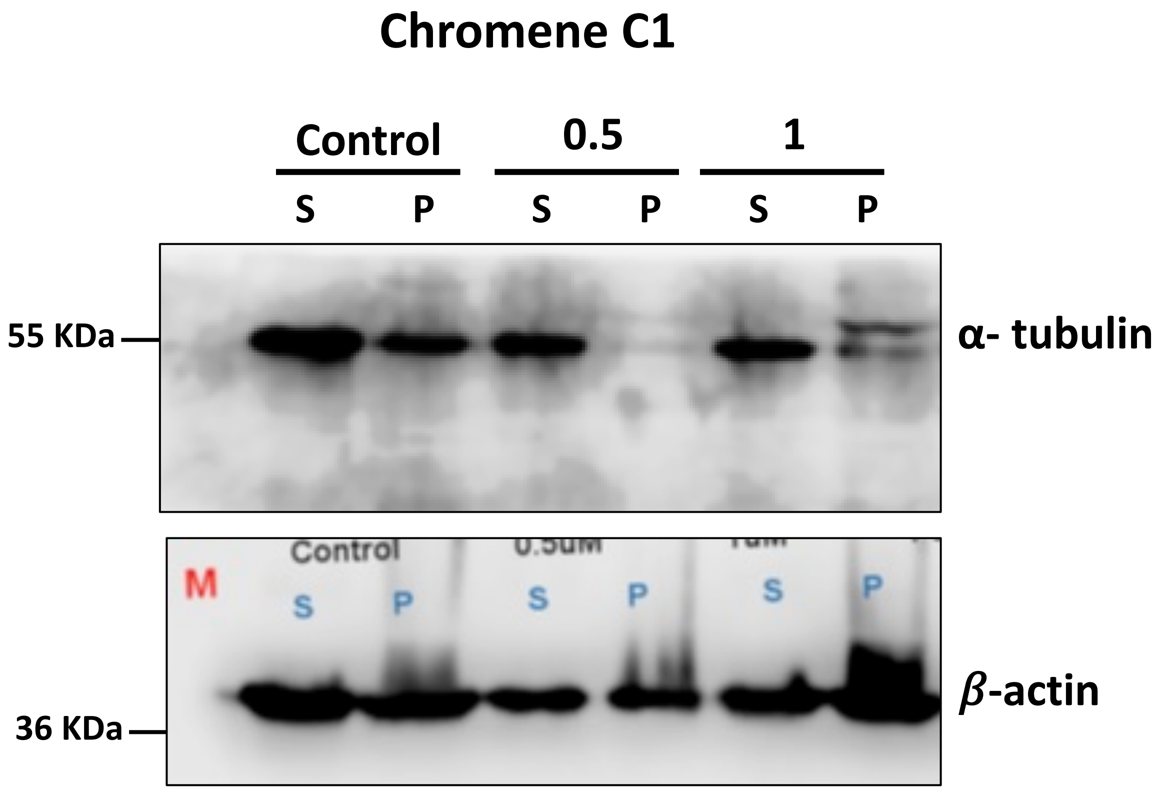


Figure 8B

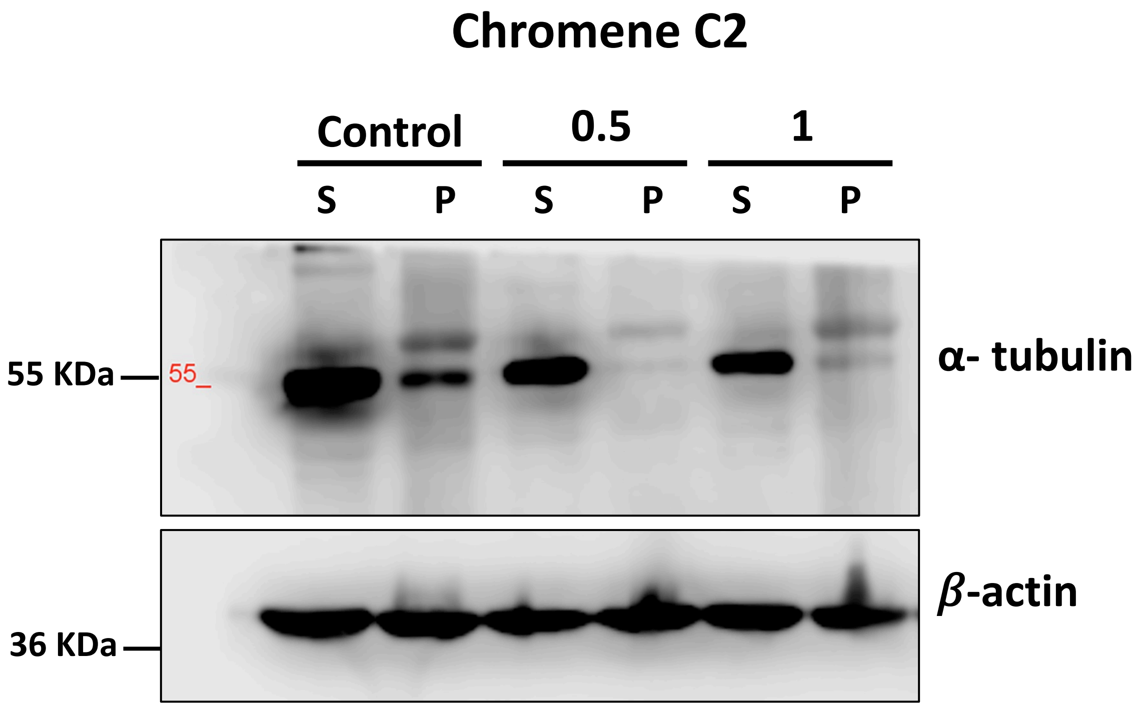


Figure 8C

# Uncropped Western blot
# (Supplementary figures)

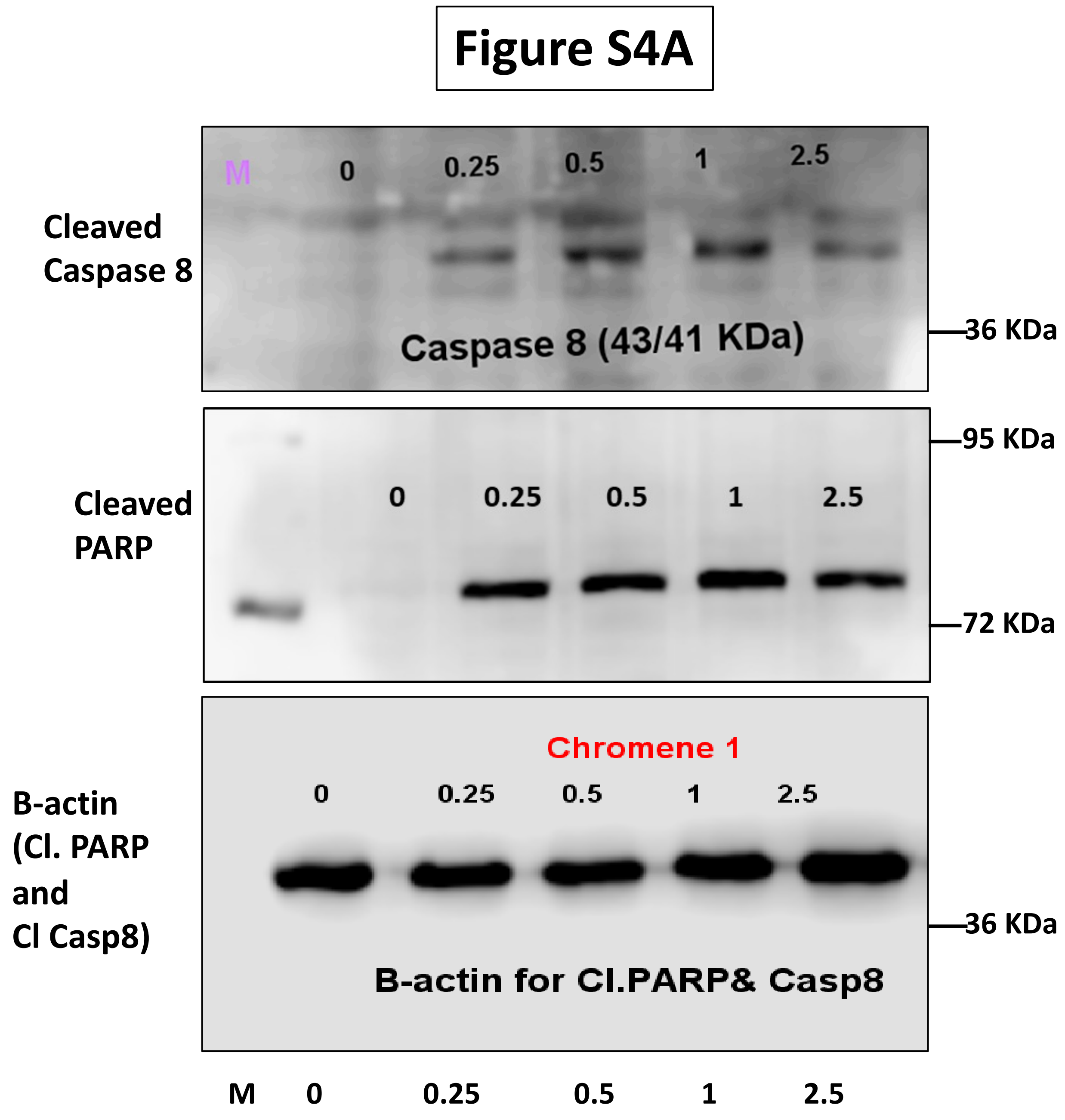

Figure S4A
Cleaved Caspase 8
M
0
0.25
0.5
1
2.5
Caspase 8 (43/41 KDa)
36 KDa
95 KDa
Cleaved PARP
72 KDa
Chromene 1
B-actin (Cl. PARP and Cl Casp8)
36 KDa
B-actin for Cl.PARP& Casp8
M 0 0.25 0.5 1 2.5


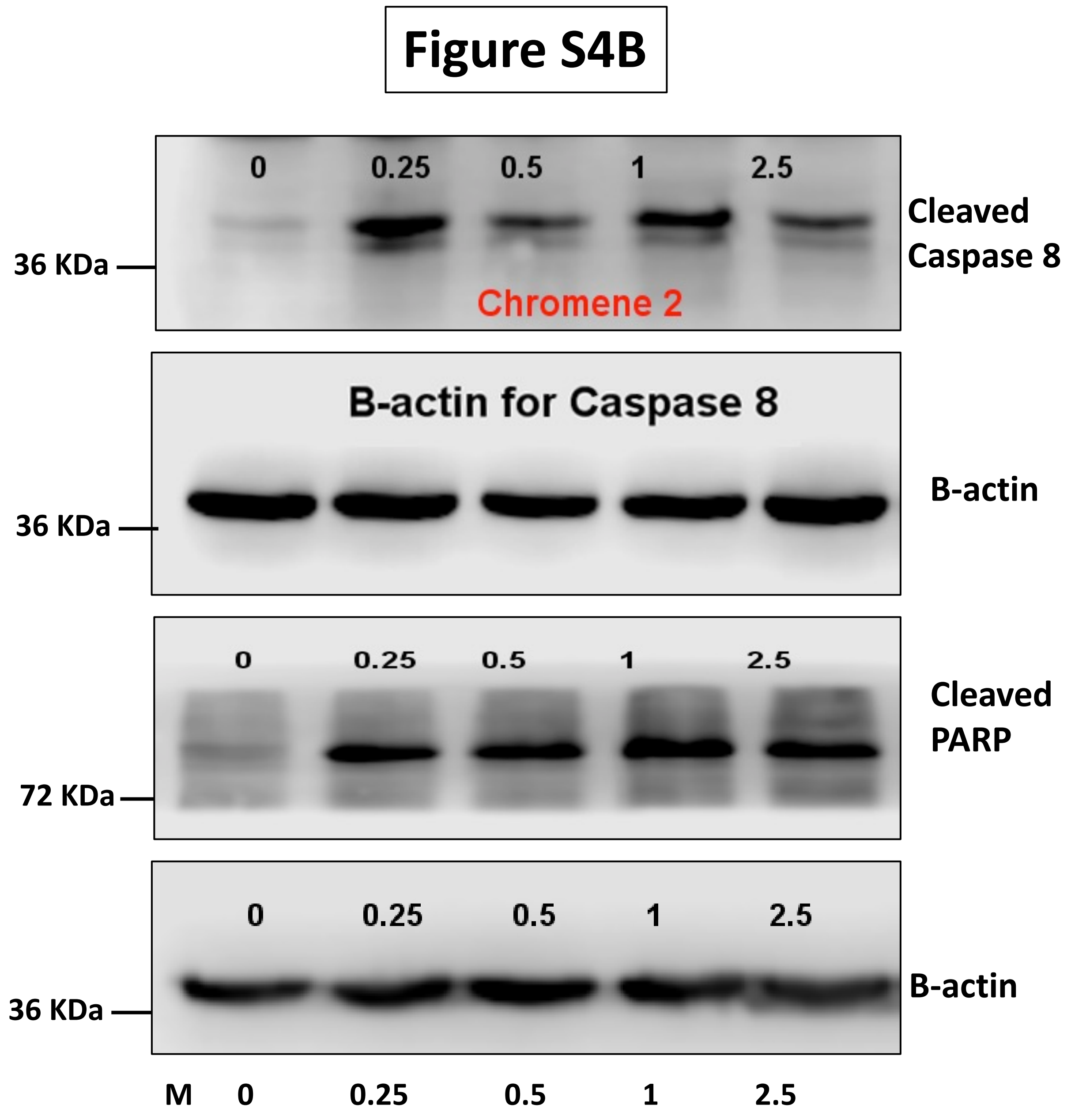

Figure S4B
0
0.25
0.5
1
2.5
Cleaved Caspase 8
36 KDa
Chromene 2
B-actin for Caspase 8
B-actin
36 KDa
Cleaved PARP
72 KDa
B-actin
36 KDa
M 0 0.25 0.5 1 2.5

# Figure S5E

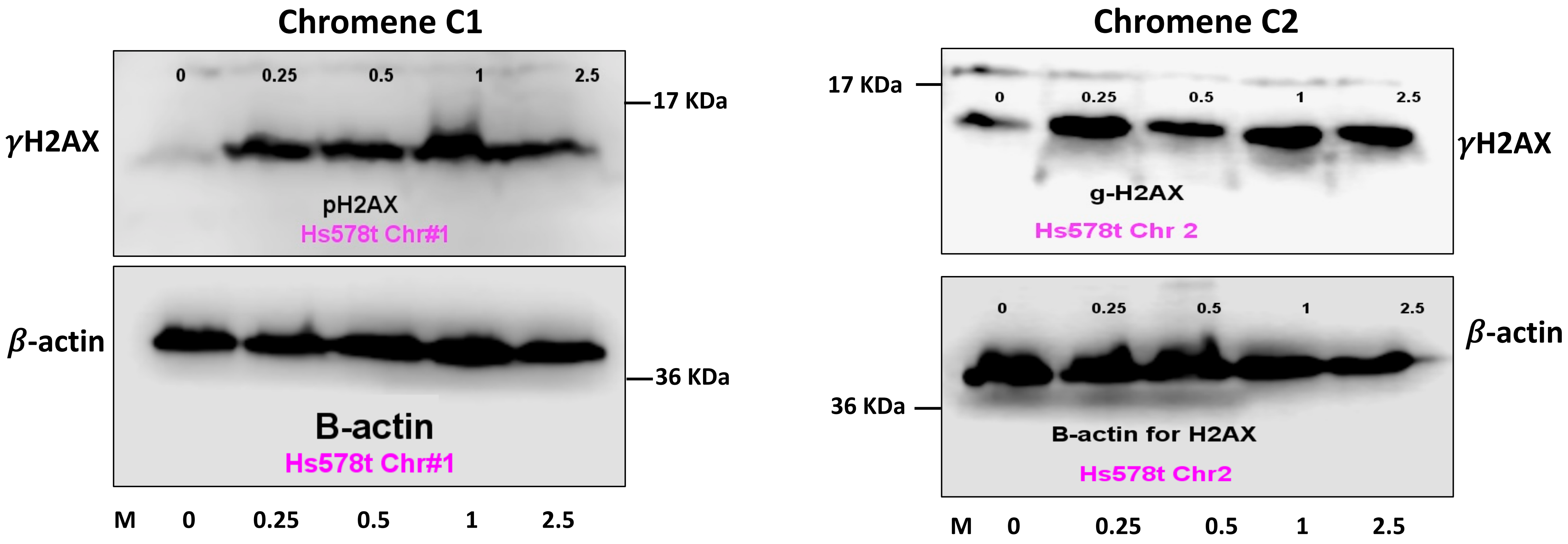
Chromene C1
γH2AX
0
0.25
0.5
1
2.5
17 KDa
pH2AX
Hs578t Chr#1
β-actin
36 KDa
B-actin
Hs578t Chr#1
M
0
0.25
0.5
1
2.5
Chromene C2
17 KDa
0
0.25
0.5
1
2.5
γH2AX
g-H2AX
Hs578t Chr 2
0
0.25
0.5
1
2.5
β-actin
36 KDa
B-actin for H2AX
Hs578t Chr2
M
0
0.25
0.5
1
2.5

# Figure S6D

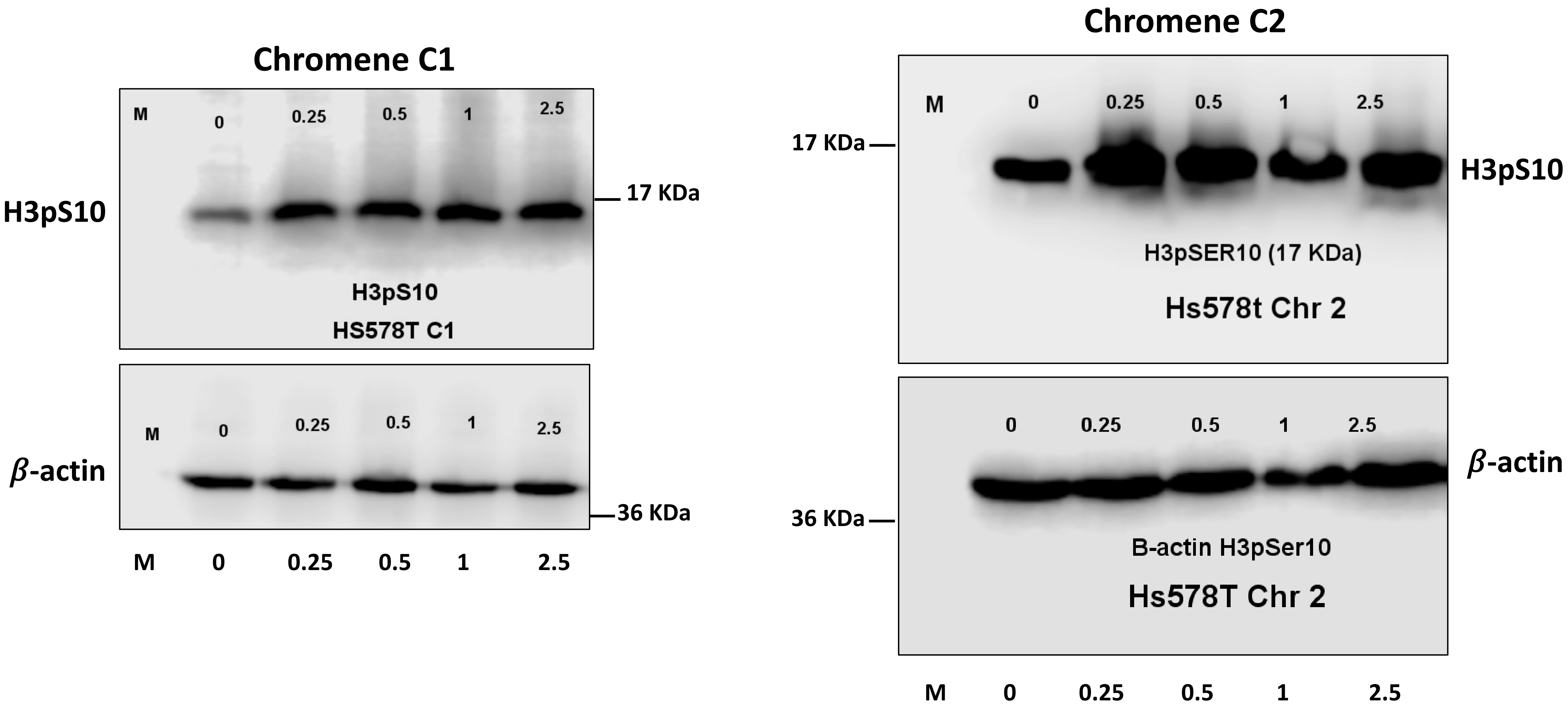

Chromene C1
H3pS10
M
0
0.25
0.5
1
2.5
17 KDa
H3pS10
HS578T C1
β-actin
36 KDa
M 0 0.25 0.5 1 2.5
Chromene C2
17 KDa
H3pS10
H3pSER10 (17 KDa)
Hs578t Chr 2
β-actin
36 KDa
B-actin H3pSer10
Hs578T Chr 2
M 0 0.25 0.5 1 2.5
