# Supplementary material for: Synthesis of New Chromene Derivatives Targeting Triple-Negative Breast Cancer Cells
Source: Cancers (Basel). 2023 May 9;15(10):2682. doi: 10.3390/cancers15102682 (PMC10216081; doi:10.3390/cancers15102682)
Supplement: Supplementary file 1 [file cancers-15-02682-s001.zip › cancers-2286253-supplementary.pdf]

File S1. Supplementary method

Synthesis of chromene derivatives

All chemicals and solvents (reagent grade from Sigma Aldrich) were used without further purification. Melting points were determined on a Stuart Scientific melting point apparatus model SMP 10 and are uncorrected. IR spectra were recorded on a Nicolet Impact 410 FT-IR spectrometer (4000-400 cm-1) as a potassium bromide (KBr) disc with 4 cm-1 resolution. 1H NMR and 13C NMR spectra were run on Varian 400 MHz and Mercury 200 MHz NMR spectrometers. The proton chemical shifts were reported in parts per million (δ ppm) and coupling constants (J) in Hertz (Hz). Letters s, d, t, m, br s refer to singlet, doublet, triplet, multiplet, and broad singlet, respectively. ESI-MS and HRMS spectra of the compounds were recorded on JEOL JMS-600H high resolution mass spectrometer.

1. Synthesis of chromene C1

A stirred mixture of 2-naphthol (1.44 g, 10 mmol), benzaldehyde (10 mmol), malonitrile (0.660g, 10 mmol), and two drops of piperidine in bench ethanol (10 mL) was refluxed in a microwave reactor at 80°C (dynamic power 25-30 W) for 5-15 minutes. The resulting solid was filtered and washed with cold ethanol several times and dried under vacuum. Recrystallization in ethanol was performed to obtain spectroscopically pure materials.

Yield: 2.77 g (93%); mp: 220-223°C; IR (KBr pellet, cm-1): 3441, 3299, 3184, 2205, 1653, 1600, 1399;1H NMR (400 MHz, DMSO-d6): δ 4.87 (s, 1H), 7.06 (d, J=8.4Hz, 1H), 7.15-7.29 (m, 6H), 7.50-7.62 (m, 3H), 7.83 (d, J= 8.4 Hz, 1H), 8.24-8.26 (m, 1H); 13C NMR (100 MHz, DMSO-d6): δ 41.4, 56.7, 118.4, 121.1, 121.2, 123.2, 124.3, 126.7, 127.1, 127.2, 127.4, 128.1, 129.2, 133.1, 143.2, 146.2, 160.6; HRMS (+ESI): calcd. for $C_{20}H_{14}N_2O$, 298.1101, found 298.1077.

2. Synthesis of Schiff base C2

C1 (298 mg, 1.0 mmol) and salicylaldehyde (134 mg, 1.1 mmol) were added to 10 mL of dry ethanol. The mixture was stirred and refluxed for 13 hours. The solution was kept in a refrigerator and the resulting yellow solids were collected, washed with cold ethanol, and dried under vacuum.

Yield: 229 mg (57%); mp: 243-249 °C, mixture of diastereoisomers; IR (KBr pellet, cm-1): 3343, 2210, 2184, 1657, 1632, 1597, 1560, 1452; 1H NMR (400 MHz, DMSO-d6): δ 4.85 (s, 1H), 5.26 (s, 1H), 3.77 (s, 3H), 4.40 (m, 0.5H), 5.25 (s, 1H), 7.05-7.07 (m, 4H), 7.17-7.35 (m, 11H), 6.96-7.18 (m, 6H), 7.49-7.62 (m, 6H), 7.82-7.89 (m, 2H),7.99-8.02 (m, 1H), 8.22 (d, J=8.8 Hz, 1H), 8.46 (d, J=8.4 Hz, 1H),9.49 (s, 1H),11.93 (s br, 1H); 13C NMR (100 MHz, DMSO-d6): δ 41.3, 42.5, 56.6, 87.1, 116.8, 117.6, 117.9, 118.35, 119.5.120.3,121.08, 121.0, 121.6, 123.2, 123.5, 124.3, 124.3,

125.5, 126.5, 126.7,127.1, 127.2, 127.4, 127.6, 128.1, 128.2, 128.9, 129.2, 129.5, 133.1, 133.5, 133.6, 136.2, 143.0, 143.1, 143.9, 146.2, 157.0, 160.6,161.3, 164.2; HRMS (+ESI): calcd for C27H18N2O2, 402.1368, found 402.1013.

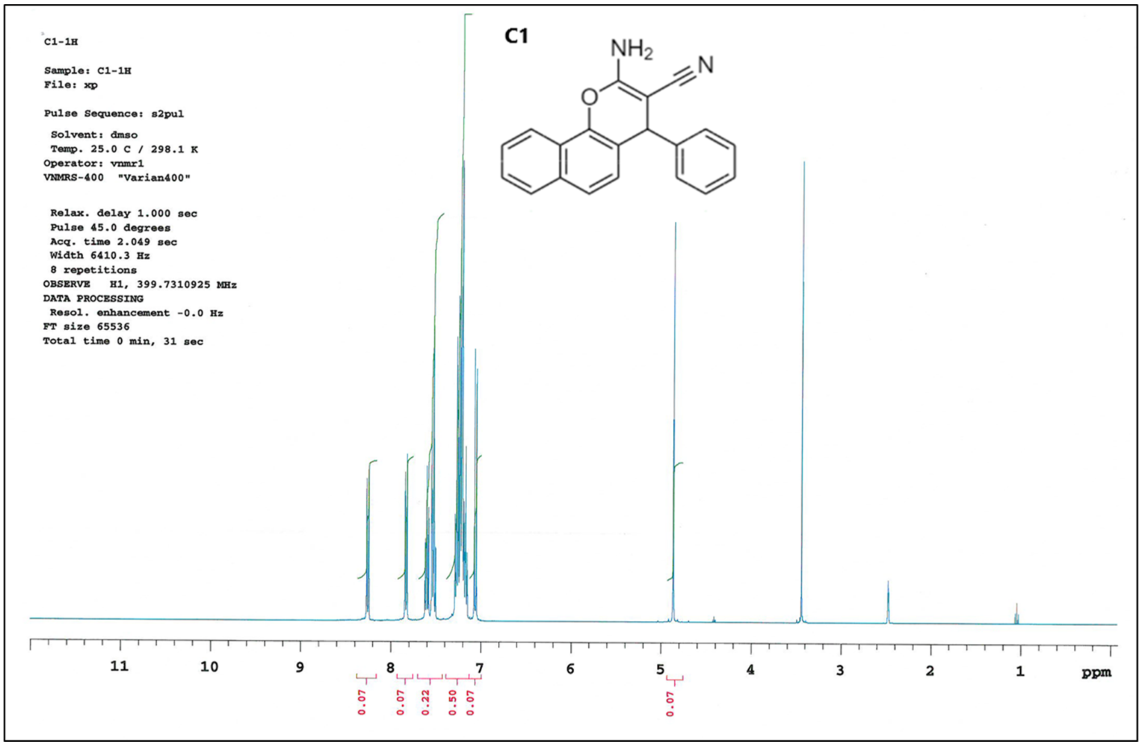

Figure S1 (a)

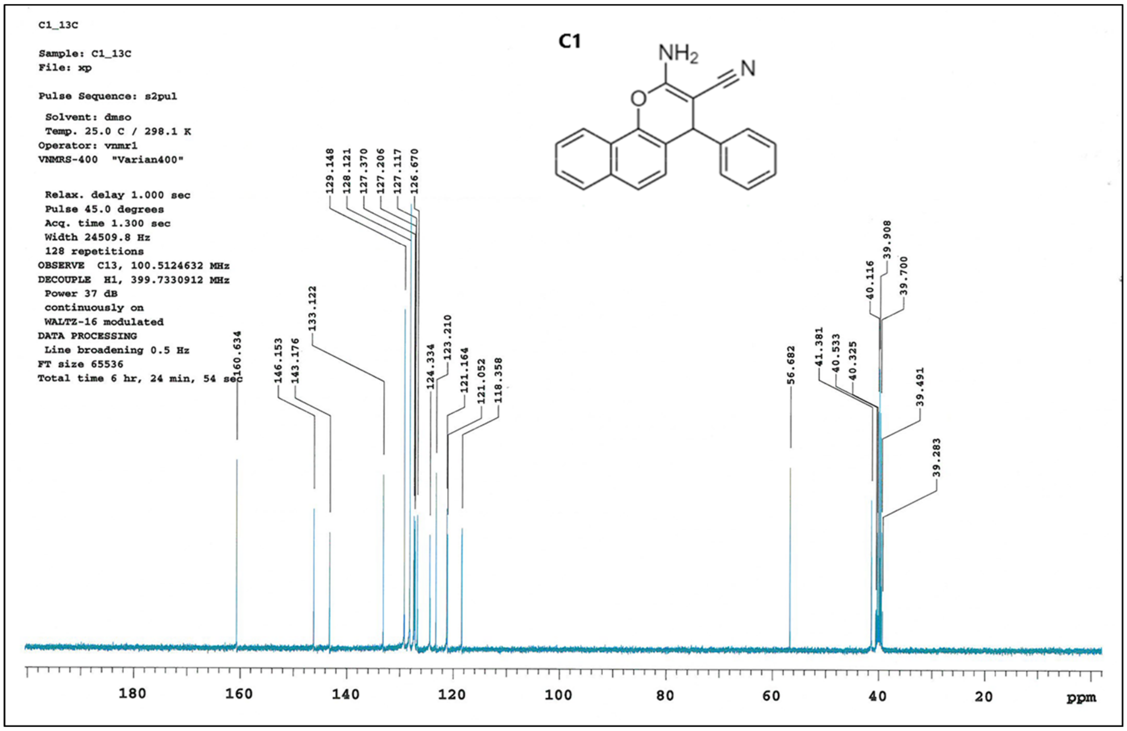

Figure S1 (b)

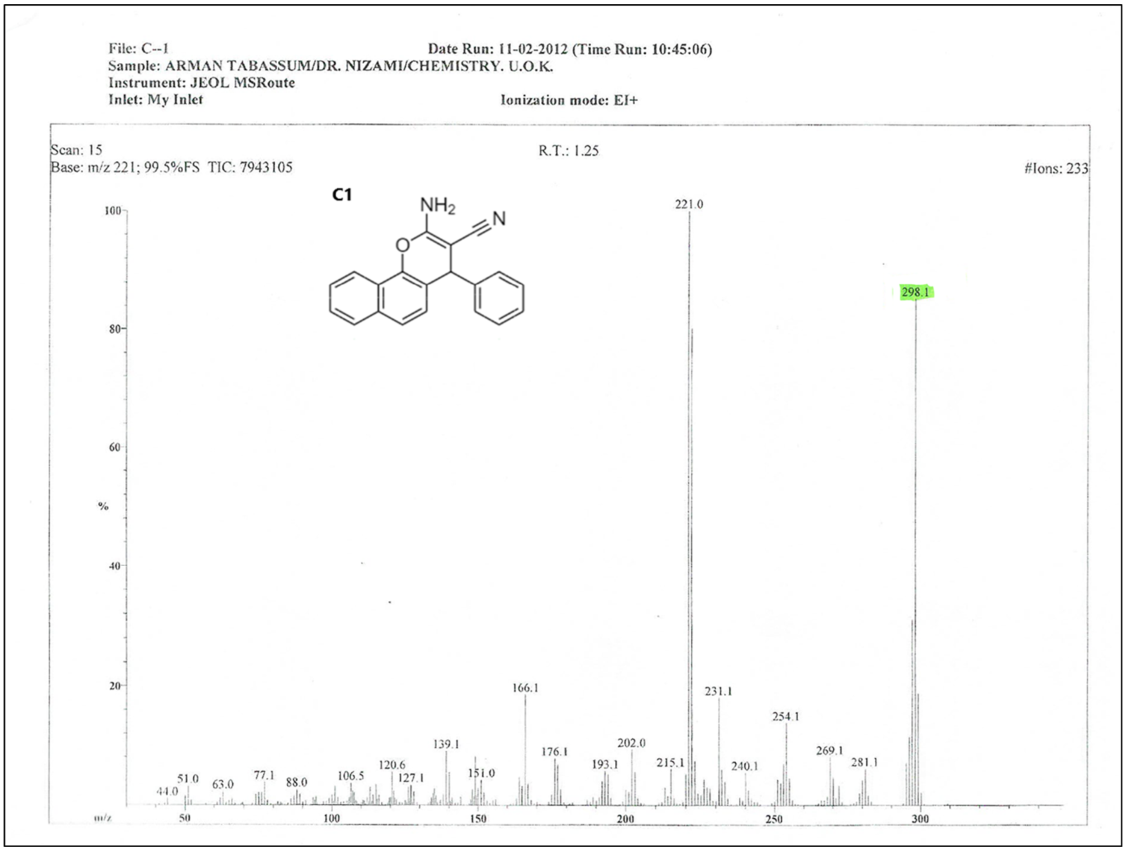

Figure S1 (c)

File: C--1
Sample: ARMAN TABASSUM/DR. NIZAMI/CHEMISTRY. U.O.K.
Instrument: JEOL MSRoute
Inlet: My Inlet
Ionization mode: EI+

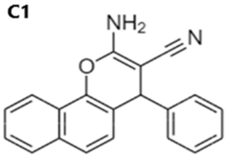

Scan: 15
R.T.: 1.25
Base: m/z 221; 99.5%FS TIC: 7943105
#Ions: 233

Threshold: 1% of Base
Displayed TIC: 7943105

Mass	%Base	Mass	%Base	Mass	%Base	Mass	%Base	Mass	%Base	Mass	%Base
43.9907	1.1	106.0150	1.3	134.5339	2.0	175.0433	1.7	220.9742	100.0	255.0875	4.5
50.0082	1.5	106.5337	3.7	135.0551	2.8	176.0606	7.8	222.0769	80.2	268.0716	1.6
51.0184	3.1	107.0276	2.4	135.5482	1.6	177.0479	6.7	223.0717	7.4	269.0764	8.1
62.0290	1.1	107.5489	2.2	138.0271	1.9	178.0710	2.7	224.0685	1.9	270.0831	4.7
63.0311	2.2	112.0286	1.3	139.0543	9.1	189.0460	1.4	225.0674	1.7	271.0512	1.2
66.0431	1.1	113.0682	3.1	140.0556	5.5	191.0609	1.3	226.0682	4.4	272.0615	3.3
74.0601	1.7	114.0584	1.8	141.0604	1.3	192.0550	4.1	227.0709	3.0	279.0604	2.0
75.0616	2.2	115.0792	3.5	144.0354	1.4	193.0516	5.7	228.0755	2.9	280.0436	4.2
76.0692	2.1	115.5640	1.0	148.0341	2.7	194.0504	5.2	231.0998	18.1	281.0695	6.1
77.0610	3.8	116.0767	1.8	148.5488	1.5	195.0511	1.2	232.0737	6.0	282.0561	1.6
86.0240	1.1	119.5810	1.3	149.0341	8.2	200.0532	2.6	233.0865	4.0	295.0372	7.1
87.0280	1.5	120.0733	1.3	149.5504	1.6	201.0676	2.3	234.1010	1.2	296.0905	11.4
88.0378	2.6	120.5665	5.6	150.0373	2.9	202.0493	9.4	238.0638	1.3	297.0611	31.1
89.0532	1.9	121.0606	2.4	151.0439	4.2	203.0687	5.6	240.0763	5.4	298.0758	85.2
93.5358	1.3	121.5832	1.3	152.0233	2.2	204.0553	1.2	241.0669	2.5	299.0922	18.8
94.5328	1.6	126.0528	3.0	163.0683	1.2	213.0435	3.0	242.0600	1.2	300.1104	2.1
98.9956	1.1	126.5572	1.2	164.0574	4.7	214.0541	1.6	251.0584	4.3		
99.9963	1.8	127.0627	3.3	165.0488	3.2	215.0670	6.1	252.0728	3.7		
101.0279	3.2	128.0483	2.3	166.0743	18.6	216.0823	1.2	253.0498	6.9		
102.0402	1.3	134.0426	1.3	167.0696	3.5	220.0573	5.2	254.0677	13.9		

Figure S1 (d)

Figure S1. Synthesis and characterization of chromene compound C1. (**a**) - 1H NMR spectrum of chromene C1 in DMSO-d6. (**b**) - 13C NMR spectrum of chromene C1 in DMSO-d6. (**c**) Low resolution mass spectrum of chromene C1. (**d**) High resolution mass spectrum of chromene C1.

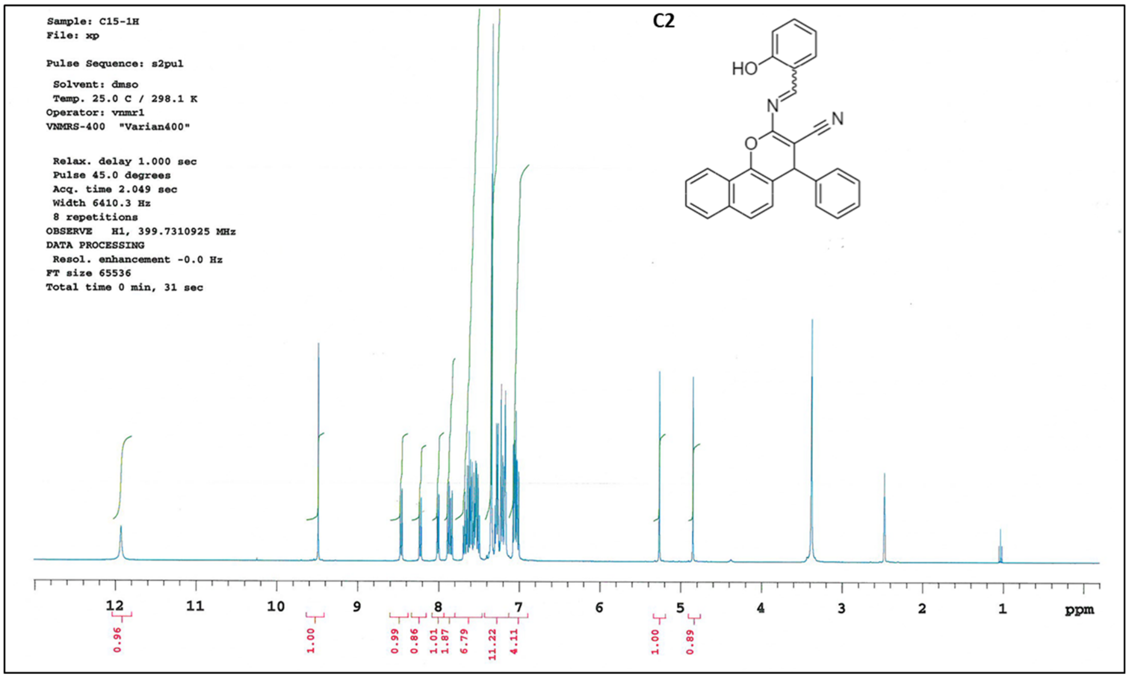

Figure S2 (a)

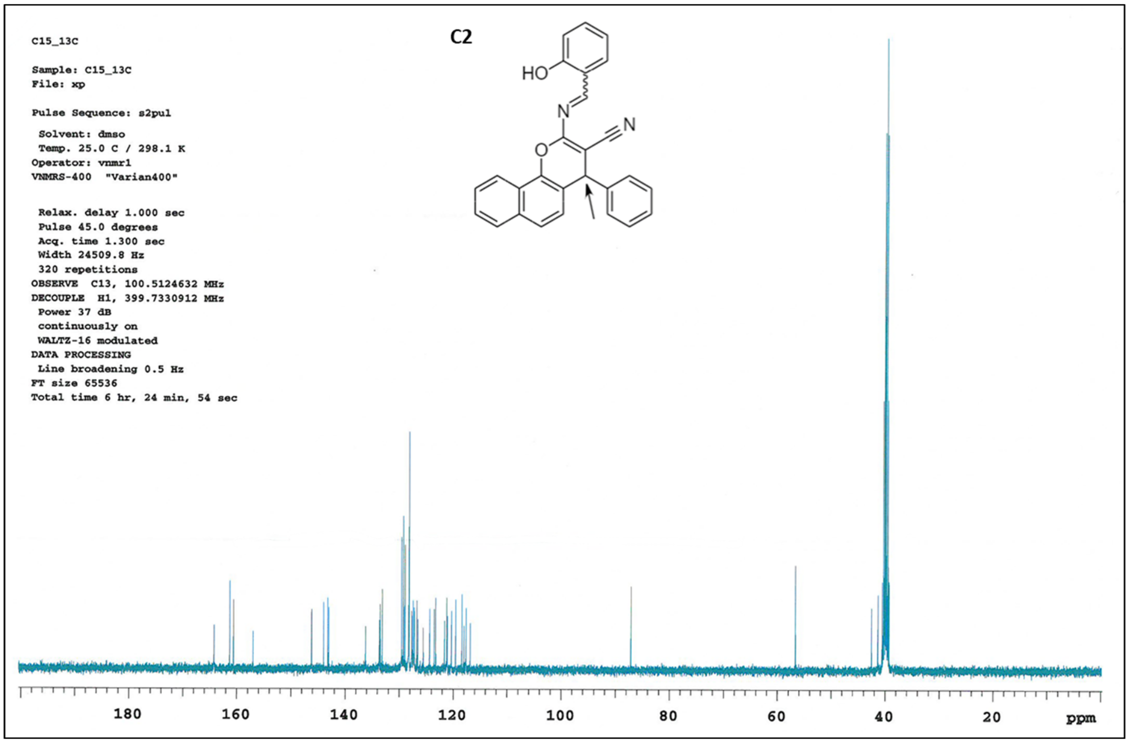

Figure S2 (b)

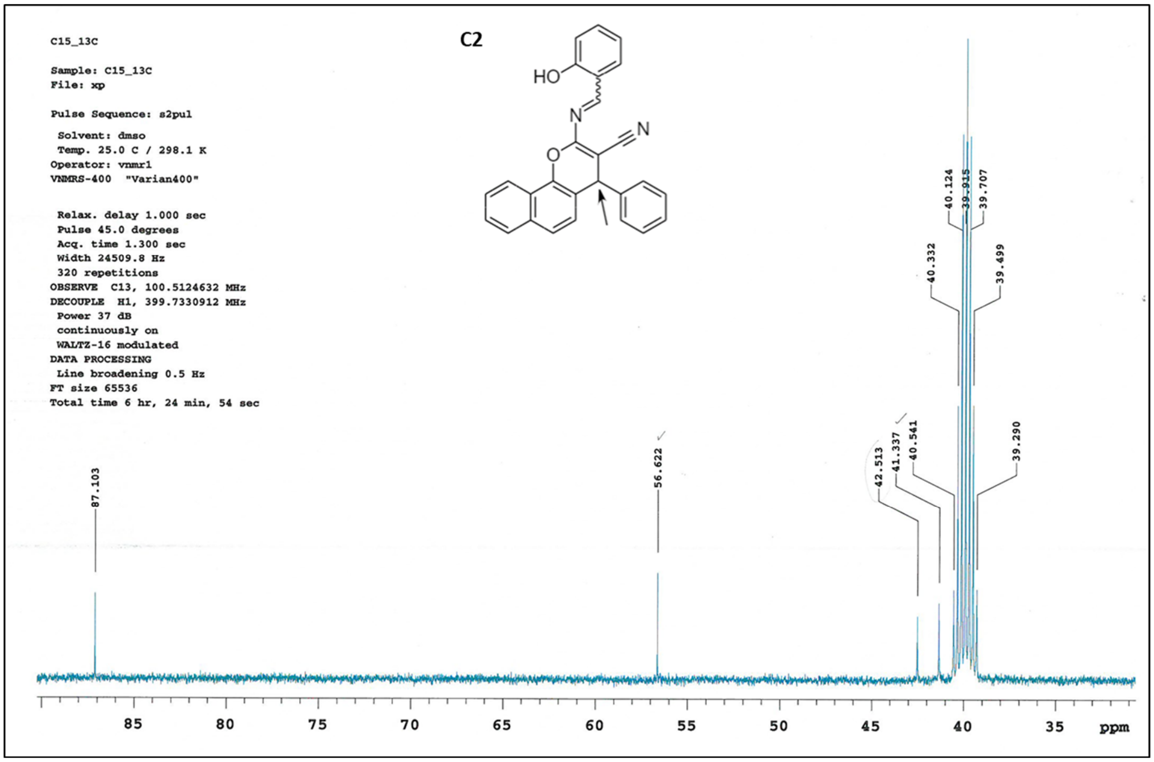

Figure S2 (c)

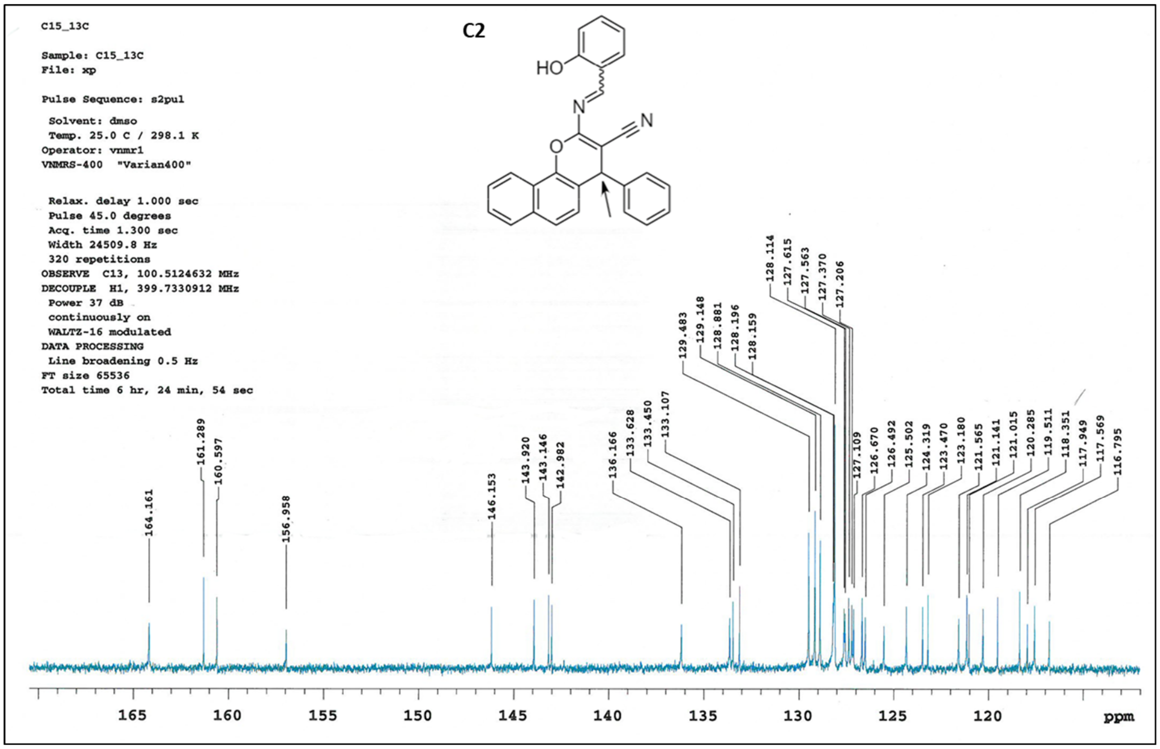

Figure S2 (d)

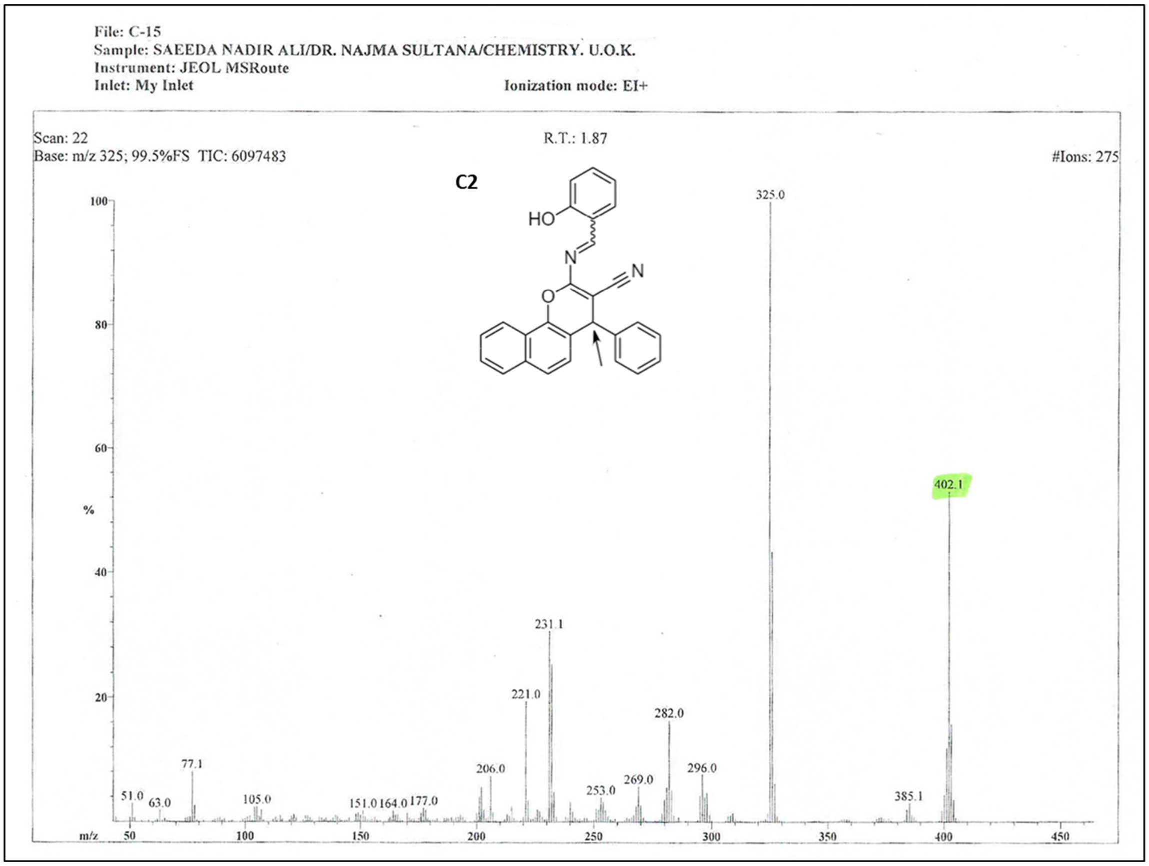

Figure S2 (e)

File: C-15
Sample: SAEEDA NADIR ALI/DR. NAJMA SULTANA/CHEMISTRY. U.O.K.
Instrument: JEOL MSRoute
Inlet: My Inlet
Ionization mode: EI+

Scan: 22
Base: m/z 325; 99.5%FS TIC: 6097483
R.T.: 1.87
#Ions: 275

Threshold: .5% of Base
Displayed TIC: 6097483

Mass	%Base	Mass	%Base	Mass	%Base	Mass	%Base	Mass	%Base	Mass	%Base
43.9739	.8	120.0733	.8	170.0326	1.4	207.0301	1.5	252.0337	1.9	307.0268	.9
50.0082	.8	120.5665	.6	171.0356	.7	213.0435	1.2	253.0498	3.9	308.0578	1.0
51.0184	2.9	121.0606	1.2	172.0414	.5	214.0179	.9	254.0285	3.2	309.0471	1.4
52.0203	.7	122.0516	.6	175.0433	.6	215.0308	2.5	255.0482	1.8	324.0265	1.3
63.0110	1.8	126.0528	1.0	176.0277	1.4	216.0460	.7	256.0304	.8	324.9916	100.0
65.0588	.5	127.0627	1.0	177.0479	2.2	220.0207	.9	264.0296	.7	326.0461	43.4
74.0384	.6	128.0483	.7	178.0379	2.0	221.0476	19.4	265.0274	.6	327.0583	6.1
75.0397	.7	132.0291	.8	179.0308	.6	222.0401	3.4	266.0270	.7	328.0279	1.0
76.0473	.9	133.0339	.6	183.0305	.5	224.0315	.6	267.0284	1.1	371.0187	.5
77.0610	8.1	135.0261	.7	186.0262	.6	225.0303	.8	268.0314	2.5	372.0054	.7
78.0587	2.6	138.0271	.6	187.0076	.7	226.0311	1.9	269.0362	5.7	373.0414	.8
88.0378	.6	139.0249	1.2	188.0254	.5	227.0337	1.7	270.0428	2.6	374.0325	.5
89.0295	.7	140.0261	.9	189.0460	.8	228.0383	.8	271.0512	.7	376.0665	.5
91.0533	.5	145.0230	.7	191.0267	.7	231.0624	30.6	279.0194	1.4	384.0386	1.9
100.0214	.5	148.0039	1.3	192.0207	.8	232.0363	25.3	280.0026	3.6	385.0863	3.0
101.0027	.8	149.0037	1.5	193.0172	1.2	233.0490	4.8	281.0285	5.5	386.0399	1.1
102.0148	.9	150.0068	1.1	194.0159	.8	234.0634	.8	282.0150	16.4	387.0424	.8
104.0302	2.3	151.0133	1.8	200.0183	1.4	238.0260	.9	283.0443	5.1	399.0363	1.8
105.0330	2.4	152.0233	.8	201.0326	4.1	239.0120	.6	284.0342	1.0	400.0568	4.3
106.0409	.9	156.0067	.7	201.5231	.9	240.0383	3.2	286.0190	.8	401.0785	11.9
106.5337	.7	162.5269	.7	202.0493	5.6	241.0288	1.6	295.0372	4.3	402.1013	53.1
107.0276	1.9	163.0364	.7	203.0335	2.1	242.0217	.7	296.0483	7.6	403.0766	15.8
113.0415	.8	164.0255	1.7	204.0200	.6	246.0168	.6	297.0189	4.0	404.1018	3.5
114.0584	.5	165.0488	1.1	205.0091	.8	247.0211	.6	298.0335	4.8	405.0791	.6
115.0792	1.1	166.0423	1.2	206.0006	7.4	251.0195	2.1	299.0498	1.2		

Figure S2 (f)

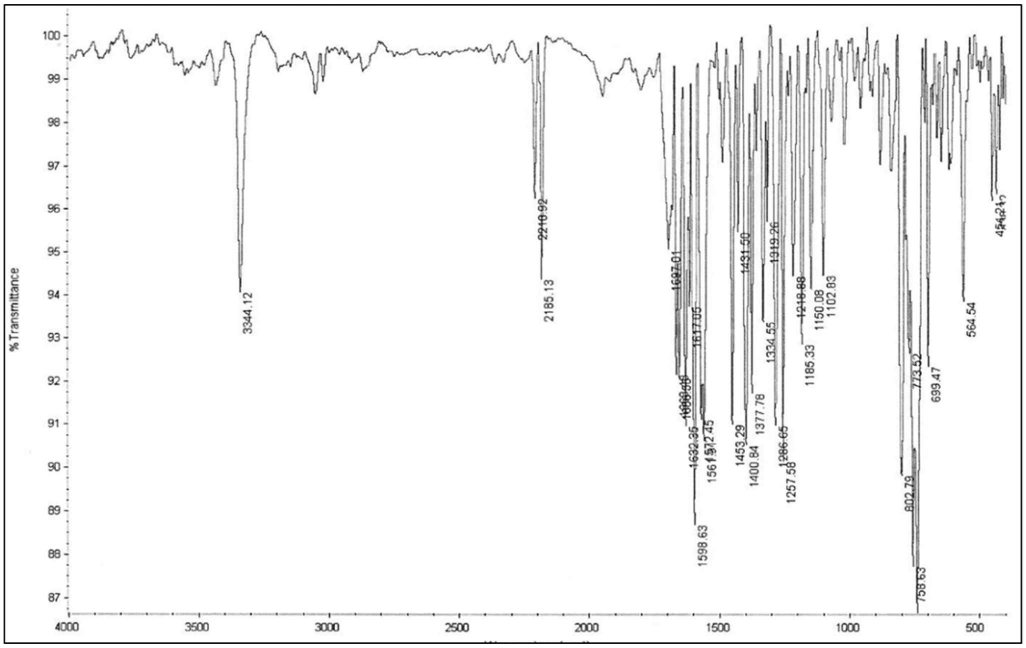

Figure S2 (g)

Figure S2. Synthesis and characterization of chromene compound C2. (**a**) 1H NMR spectrum of chromene C2 in DMSO-d6. (**b**) - Full 13C NMR spectrum of chromene C1 in DMSO-d6. (**c**) Expanded 13C NMR spectrum of chromene C2 in DMSO-d6. (**d**) Expanded 13C NMR spectrum of chromene C2 in DMSO-d6 (**e**) Low resolution mass spectrum of chromene C2. (**f**) High resolution mass spectrum of chromene C2. (**g**) Infrared spectrum of chromene C1

A.

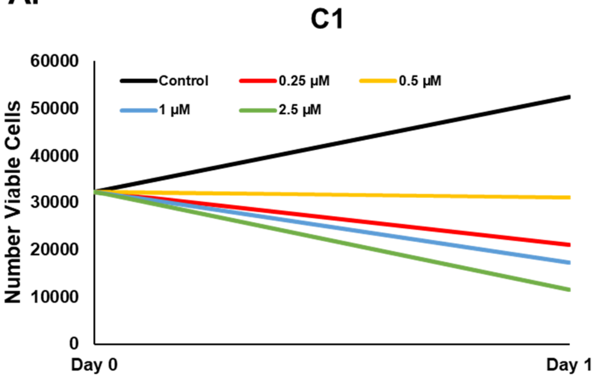

		Control	0.25 µM	0.5 µM	1 µM	2.5 µM
Viable Cells (x 10^3)	Day 0	32.4 ± 1.6				
	Day 1	52.4 ± 5	19 ± 2.6	20 ± 2.8	17 ± 2.6	11 ± 4.1

B.

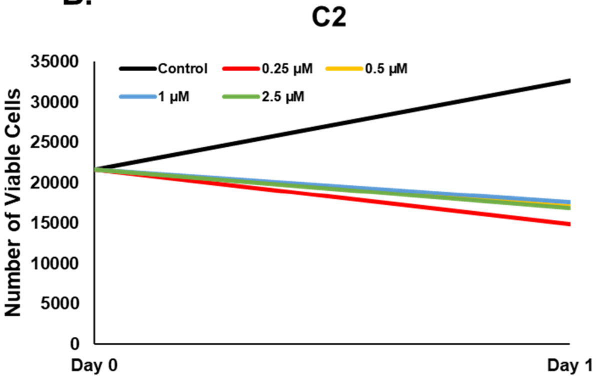

		Control	0.25 µM	0.5 µM	1 µM	2.5 µM
Viable Cells (x 10^3)	Day 0	43.4 ± 2.6				
	Day 1	64.12 ± 3.1	29.8 ± 2.4	34.2 ± 3.6	35.2 ± 1.4	33.6 ± 1.42

C.

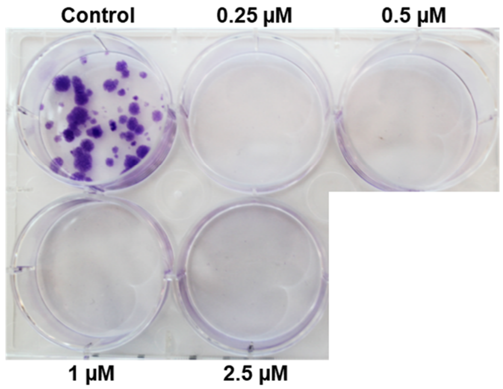

D.

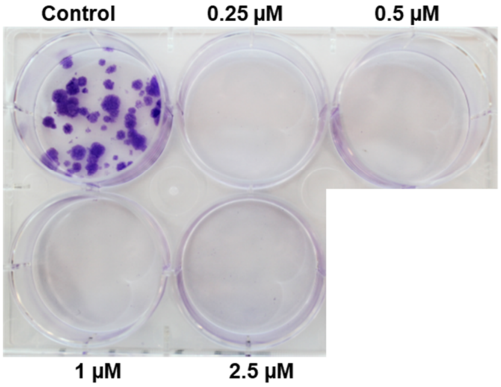

Figure S3. Chromene C1 and C2 induces cellular mortality and inhibit anchorage-dependent colony formation of Hs578T triple negative breast cancer cells. (**A-B**) Determination of cellular viability through cell counting. Hs578T cells were treated with vehicle (0.1% DMSO) and the indicated concentrations of chromene **C1 (A)** or **C2 (B)** for 24 and cell viability was monitored using the Muse cell analyzer as described in materials and methods. Data represent the mean ± SEM of three independent experiments. (**C-D**) Inhibition of colony growth Hs578T colony growth. Hs578T cells were cultured for 3 days before adding freshly prepared growth medium with or without various concentrations of chromene **C1** or **C2**.

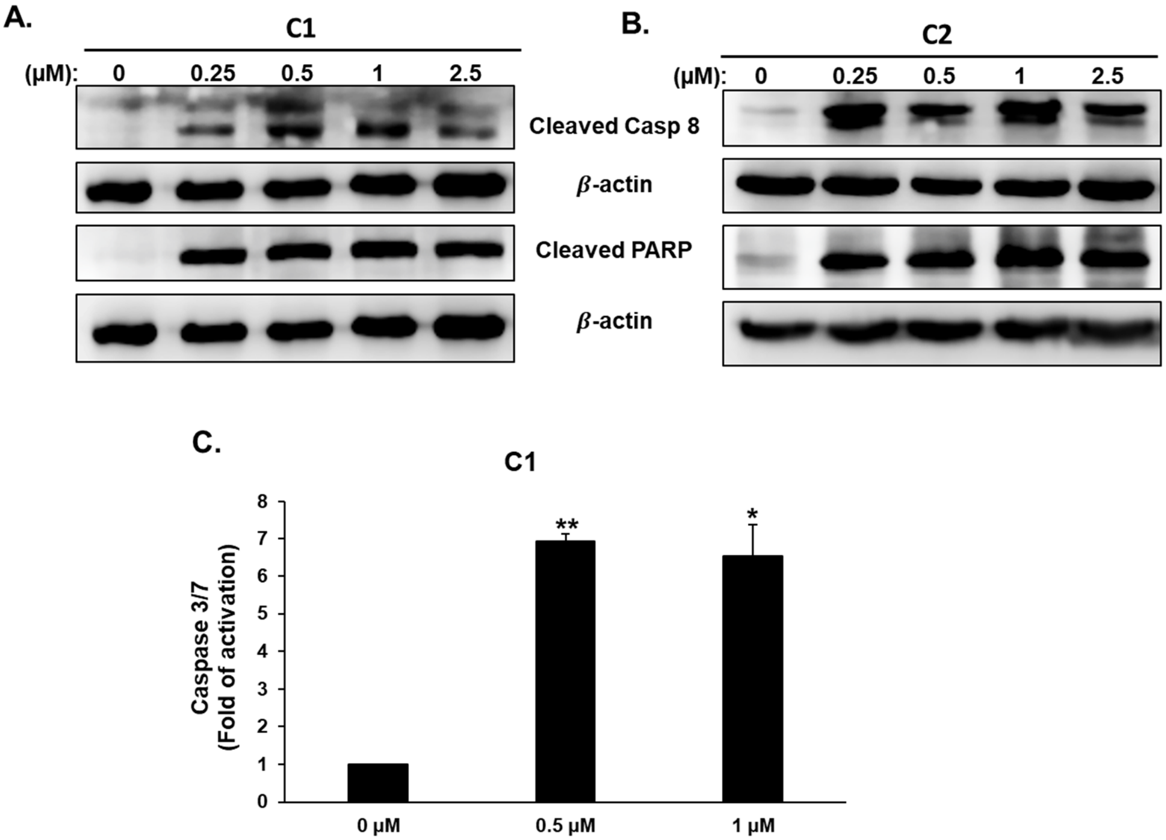

Figure S4. Chromene C1 and C2 activate the extrinsic apoptotic pathway in Hs578T cells. (A-B) Western blot analysis of caspase-8 and PARP cleavage in Hs578T cells treated with or without the indicated concentrations of **C1** (**A**) or **C2** (**B**). **(C)** Stimulation of caspase 3/7activity in Hs578T cells after exposure to **C1** for 24 hrs. The relative caspase 3/7 activity was normalized to the number of viable cells per well and is expressed as fold of activation compared to the control cells. Data represent the mean ± SEM of 3 independent experiments carried out in triplicate. (*) Significantly different at $P < 0.05$, **Significantly different at $P < 0.005$.

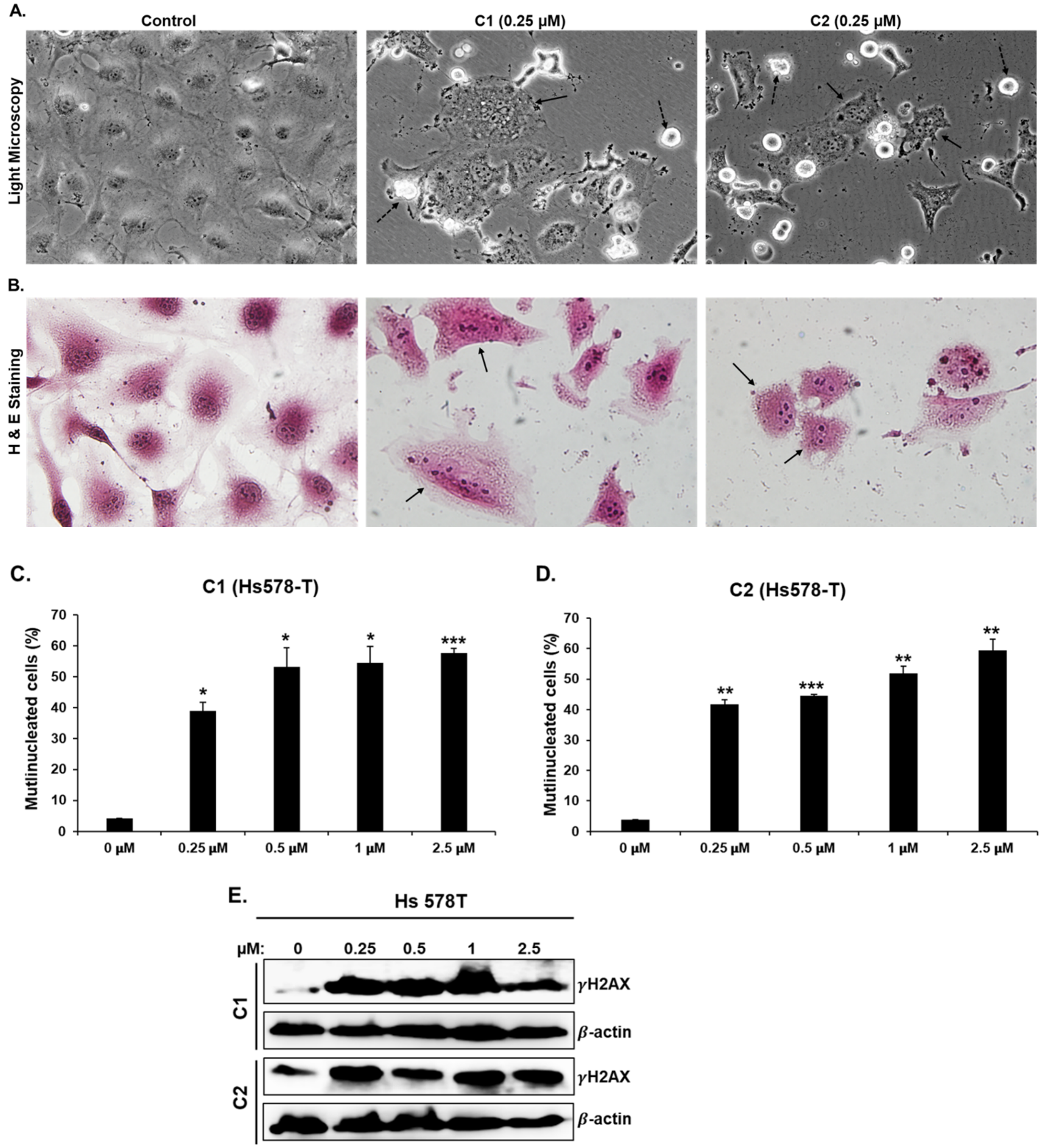

Figure S5. Chromene C1 and C2 induce multinucleation in Hs578T cells. (**A**) Morphological changes observed in Hs578T cells treated for 24 hrs with or without 0.25 mM of **C1** or **C2**. Cells were observed under EVOS XL Core Cell Imaging System (Life Technologies) at 400X. (**B**) Treated Hs578T under the same condition as in section (A) above were subjected to Hematoxylin-Eosin staining. Cells were photographed under Olympus light microscope at 200X equipped with DP74. **(C-D)** quantification of multinucleated cells treated Hs578T with **C1 (C)** or **C2 (D).**

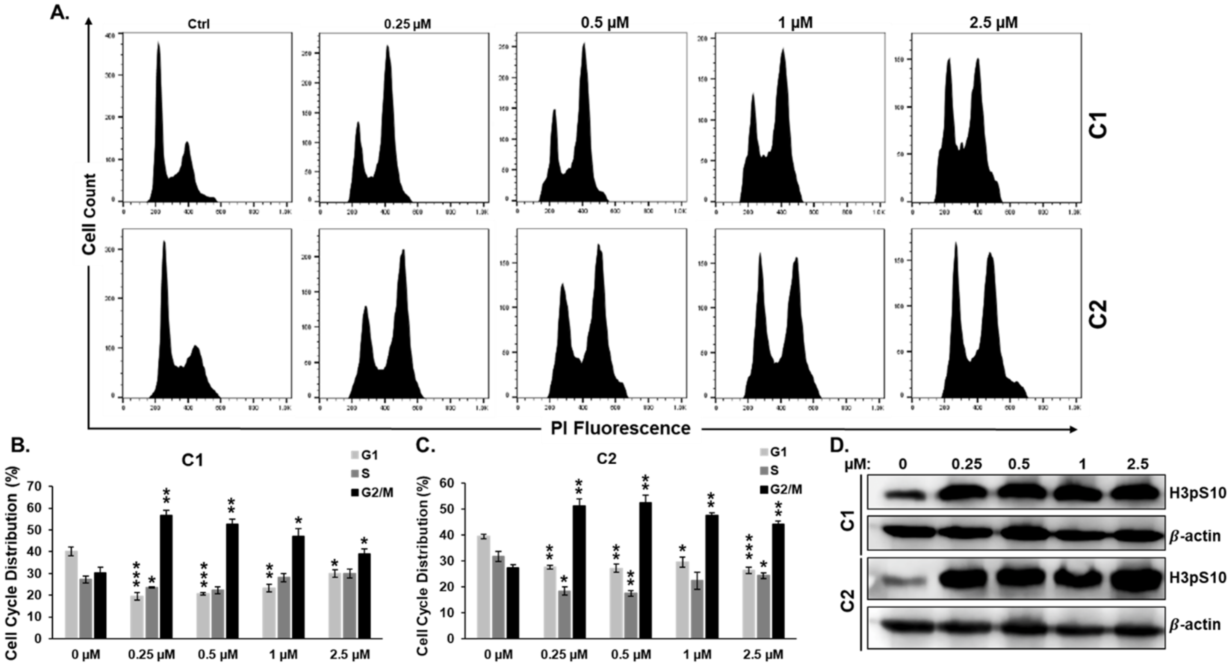

Figure S6. C1 and C2 induce a mitotic arrest in Hs578T cells. (**A-C**) Cell cycle distribution analysis in Hs578T cells treated with and without **C1** or **C2** for 24 hrs. Values are represented as mean ± SEM of 3 independent experiments carried out in duplicate (*p < 0.05, **p < 0.005). (**D**) Western blot analysis of H3pSer10, a marker of M phase, in Hs578T cells treated with or without **C1** or **C2**.

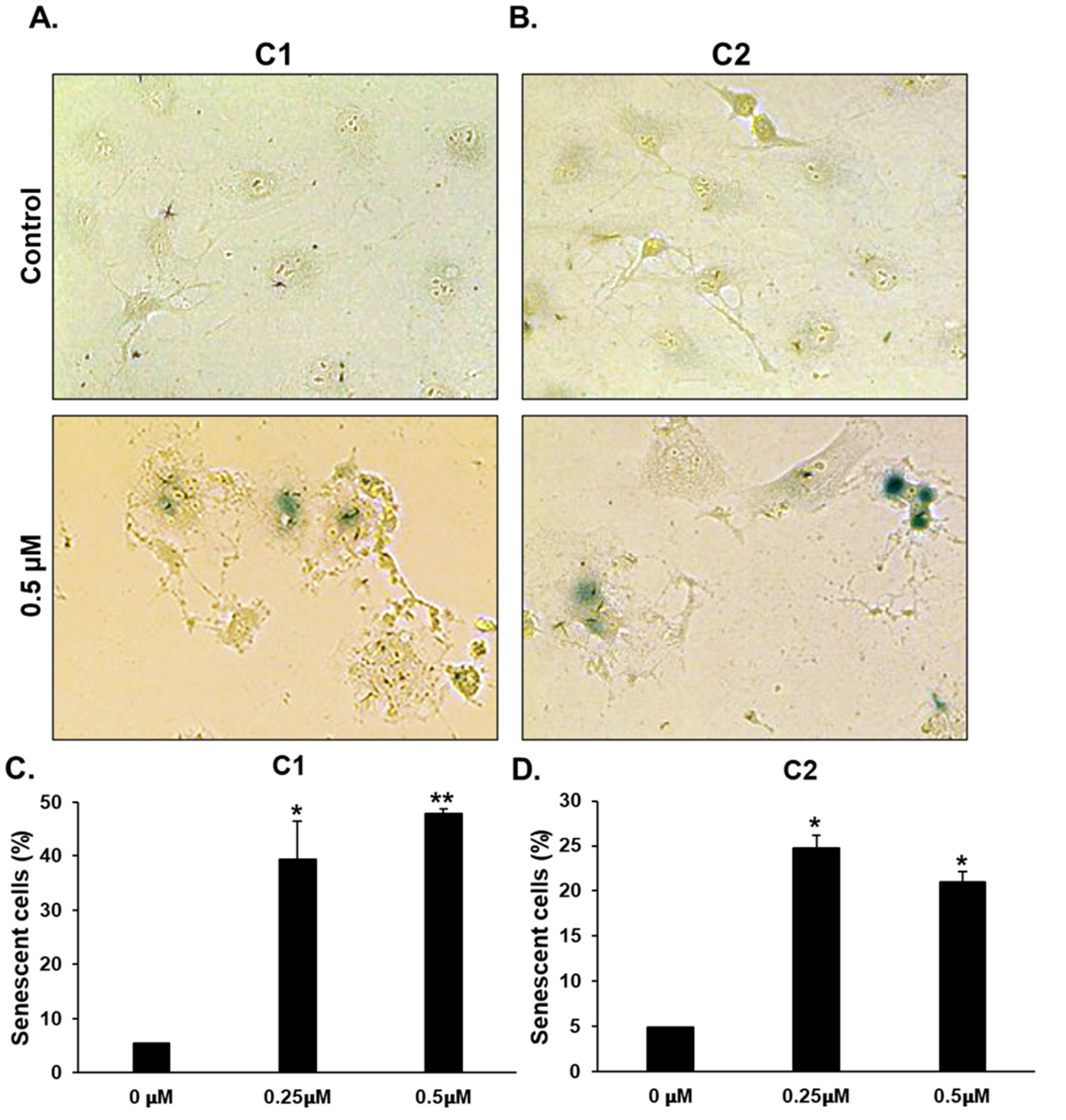

Figure S7. Induction of senescence in C1- and C2-treated Hs578T cells. (**A-D**) Detection of senescence in **C1**- and **C2**-treated cells. Hs578T cells were incubated with or without chromene **C1** or **C2** for 48 hrs and stained for SA-β-Galactosidase activity to detect senescence. Values are represented as mean ± SEM of 3 independent experiments (**p < 0.05*, ****p < 0.001*). (**E-F**) Upregulation of p16 and p21 protein levels. Cells were treated with or without the indicated concentrations of **C1** (**E**) or **C2 (F)** for 24 hrs and the protein level of p16, and p21 was examined by western blotting.

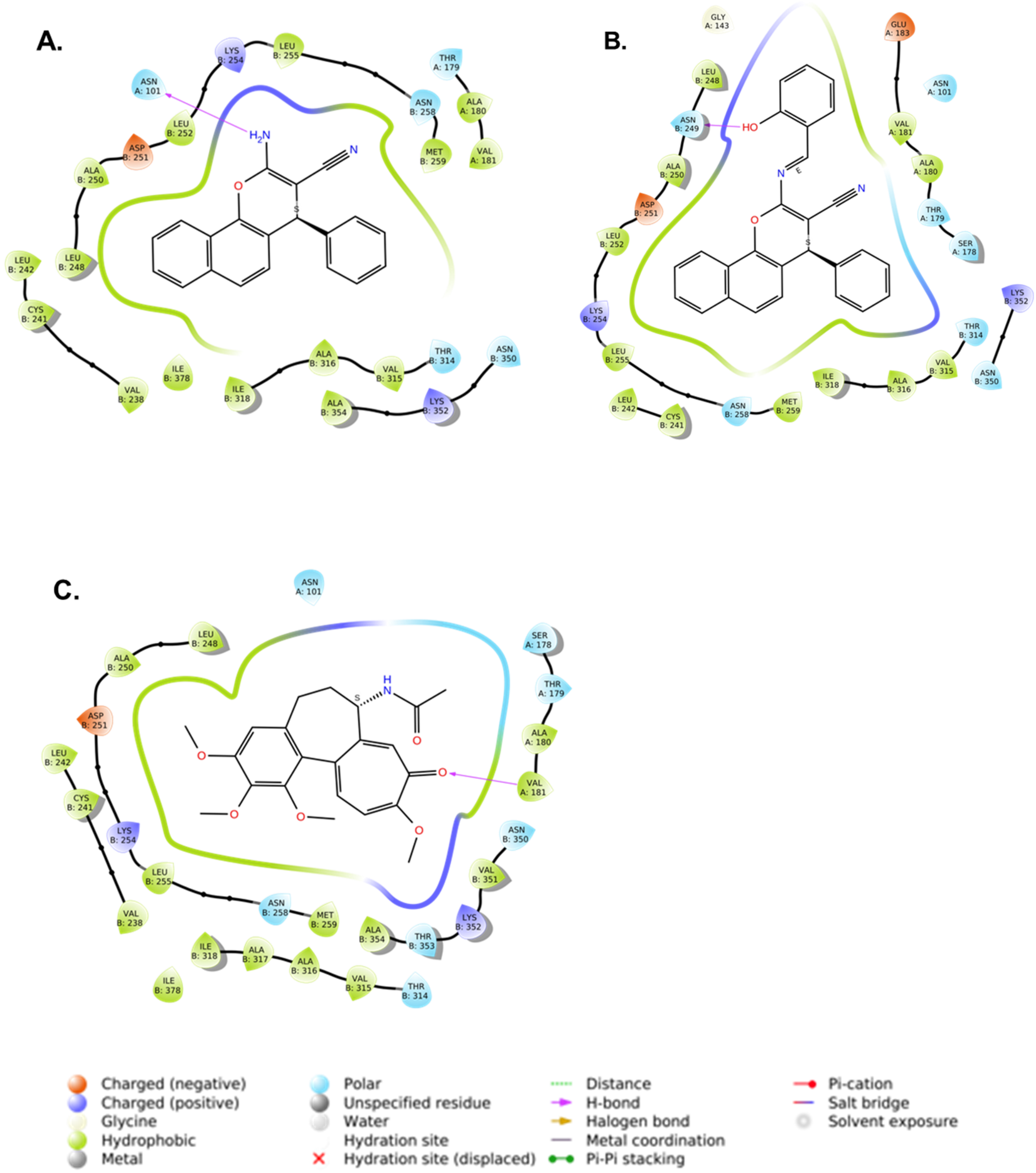

Figure S8. Ligand interaction diagram showing residues that interact with docked (**A**) C1 and (**B**) C2 and (**C**) co-crystallized colchicine. Chain A is α-tubulin and chain B is β-tubulin. Chain A residues are indicated by the letter “A” near the residue name and chain B residues indicated by the letter “B”.
